# Deep sequencing-based microRNA expression signatures in head and neck squamous cell carcinoma: dual strands of pre-*miR*-150 as antitumor miRNAs

**DOI:** 10.18632/oncotarget.16327

**Published:** 2017-03-17

**Authors:** Keiichi Koshizuka, Nijiro Nohata, Toyoyuki Hanazawa, Naoko Kikkawa, Takayuki Arai, Atsushi Okato, Ichiro Fukumoto, Koji Katada, Yoshitaka Okamoto, Naohiko Seki

**Affiliations:** ^1^ Department of Functional Genomics, Chiba University Graduate School of Medicine, Chuo-Ku, Chiba, Japan; ^2^ Department of Otorhinolaryngology/Head and Neck Surgery, Chiba University Graduate School of Medicine, Chiba, Japan; ^3^ Moores Cancer Center, University of California, San Diego, La Jolla, CA, USA

**Keywords:** microRNA, miR-150, ITGA3, ITGA6, TNC

## Abstract

We adopted into RNA-sequencing technologies to construct the microRNA (miRNA) expression signature of head and neck squamous cell carcinoma (HNSCC). Our signature revealed that a total of 160 miRNAs (44 upregulated and 116 downregulated) were aberrantly expressed in cancer tissues. Expression of *miR-150-5p* (guide strand miRNA) and *miR-150-3p* (passenger strand miRNA) were significantly silenced in cancer tissues, suggesting both miRNAs act as antitumor miRNAs in HNSCC cells. Ectopic expression of mature miRNAs, *miR-150-5p* and *miR-150-3p* inhibited cancer cell aggressiveness. Low expression of *miR-150-5p* and *miR-150-3p* predicted significantly shorter overall survival in patients with HNSCC (*P* = 0.0091 and *P* = 0.0386) by Kaplan–Meier survival curves analyses. We identified that integrin α3 (*ITGA3*), integrin α6 (*ITGA6*), and tenascin C (*TNC*) were coordinately regulated by these miRNAs in HNSCC cells. Knockdown assays using siRNAs showed that *ITGA3, ITGA6 and TNC* acted as cancer promoting genes in HNSCC cells. Moreover, *ITGA3, ITGA6*, and *TNC* alterations were associated with significantly poorer overall survival (*P* = 0.0177, *P* = 0.0237, and *P* = 0.026, respectively). Dual strands of *pre-150* (*miR-150-5p* and *miR-150-3p*) functioned as antitumor miRNAs based on the miRNA expression signature of HNSCC. Identification of antitumor *miR-150*-mediated RNA networks may provide novel insights into pathogenesis of HNSCC.

## INTRODUCTION

Despite advances in combination therapies involving surgery, radiotherapy, chemotherapy, and currently available molecular-targeted therapy for head and neck squamous cell carcinoma (HNSCC), the survival rate of patients with this disease has not improved [1–3]. HNSCC develops in the mucous membranes of the nasopharynx, oral cavity, oropharynx, larynx, and hypopharynx [4]. Among patients with HNSCC, hypopharyngeal squamous cell carcinoma is one of the most aggressive malignancies and has an extremely low survival rate due to the high rates of locoregional recurrence and distant metastasis [1, 5]. Control of cancer recurrence and metastasis may lead to improvement of HNSCC disease prognosis. Therefore, it is needed to improve our understanding of the molecular pathogenesis of HNSCC aggressiveness using current genomic-based strategies.

MicroRNAs (miRNAs) act as fine-tuners that regulate the expression control of protein-coding/protein-noncoding genes by sequence depending manner [6]. A vast extent of studies have reported that dysregulation of miRNA expression is involved in the pathogenesis of human cancers, including HNSCC [7, 8]. As a unique nature of miRNA biogenesis, a single miRNA can control thousands of target RNAs [9]. Thus, identification of novel cancer networks mediated by dysregulated miRNAs may provide insight into the pathogenesis and treatment of cancer.

Current advanced genome-based technologies can identify abnormally expressed miRNAs in various types of cancer cells [10]. To identify differentially expressed miRNAs, we used clinical specimens to establish microarray-based, polymerase chain reaction (PCR)-based, and deep sequencing-based miRNA expression signatures [11–19]. From the HNSCC miRNA expression signature, we have identified the *miR-29*-family, *miR-218*, *miR-451a*, and the *miR-26*-family acted as antitumor miRNAs and their miRNAs mediated metastasis-promoting pathways [13, 14, 20–22].

In this study, we newly constructed the RNA sequencing-based miRNA expression signature of HNSCC using laryngeal and hypopharyngeal clinical specimens. Our present data showed that 160 miRNAs (44 upregulated and 116 downregulated) were aberrantly expressed in cancer tissues. Among the downregulated miRNAs, we focused on the dual strands of *pre-miR-150*, i.e., *miR-150-5p* and *miR-150-3p*, in the HNSCC signature. Both of these miRNAs were markedly reduced in HNSCC tissues, indicating that these miRNAs may act as antitumor miRNAs in HNSCC cells. However, the roles of these miRNAs and their targets in HNSCC cells are still unclear.

It is generally accepted in miRNA biogenesis that processing of the pre-miRNA through Dicer1 generates a miRNA duplex (a passenger strand and a guide strand). It is thought that guide strand of miRNA is incorporated into the RNA-induced silencing complex (RISC) to target messenger RNAs, whereas the passenger strand of miRNA is degraded and has not functioned regulatory activity in cells [23–25]. The aim of this study was to investigate the antitumor functions of both strands of pre-*miR-150* (*miR-150-5p* and *miR-150-3p*) and to identify their targeted oncogenic genes and pathways in HNSCC cells. Elucidation of antitumor miRNA-mediated cancer networks may provide novel insights into the molecular pathogenesis of HNSCC.

## RESULTS

### Sequencing of small RNAs and construction of the miRNA expression signature of HNSCC

To prepare the miRNA expression signature of HNSCC, we performed small RNA sequencing of 6 HNSCC samples (Table 1). Initially, 12,180,452 to 24,845,427 raw sequence reads were analysed for the small RNA libraries (Supplementary Table 1). After filing and trimming of the sequenced reads, from 5,690,747 to 15,951,587 locations aligned uniquely and multiply matched reads were obtained (Supplementary Table 2). All of the sequenced reads (larger than 19 nucleotides) were assigned on the human genome, and these human genome-matched sequenced reads were divided into small RNAs according to their biological features (Supplementary Table 3).

**Table 1:** Clinical features of 22 HNSCC patients

No.	Age	Sex	Location	T	N	M	Stage	Differentiaion	remarks
1	66	M	hypopharynx	2	2c	0	IVa	moderate	deep sequencing
2	66	M	hypopharynx	4b	2c	0	IVb	moderate	deep sequencing
3	45	M	hypopharynx	4a	2c	0	IVa	moderate	deep sequencing
4	75	M	hypopharynx	4a	2c	0	IVa	well	deep sequencing
5	82	M	larynx	4a	0	0	IVa	moderate	deep sequencing
6	50	M	larynx	4a	2b	0	IVa	moderate	deep sequencing
7	58	M	hypopharynx	4a	0	0	IVa	well	IHC staining
8	76	M	hypopharynx	4a	1	0	IVa	well	
9	66	M	hypopharynx	4a	2c	0	IVa	well	
10	74	M	hypopharynx	4a	2c	0	IVa	poor	
11	69	M	larynx	3	0	0	III	well	
12	85	M	larynx	3	2b	0	IVa	moderate	
13	70	M	larynx	4a	1	0	IVa	well-moderate	
14	84	M	larynx	4a	0	0	IVa	moderate	
15	74	M	tongue	1	0	0	I	well	
16	66	M	tongue	2	0	0	II	moderate	IHC staining
17	73	M	tongue	3	1	0	III	poor	
18	72	M	tongue	4a	2b	0	IVa	moderate	
19	83	M	oral floor	2	1	0	III	well	
20	77	M	oral floor	2	2b	0	IVa	moderate	
21	68	F	oral floor	4a	1	0	IVa	well	
22	69	M	orophalynx	1	0	0	I	well	

In this study, a total of 116 downregulated miRNAs and 44 upregulated miRNAs were detected from aligned reads using R program (Table 2 and Supplementary Tables 4A and 4B).

**Table 2:** Downregulated miRNAs identified by deep sequencing of HNSCC clinical specimens

MicroRNA	Accession No.	Location	Log2 fold change	FDR	Normalized read count (Log2)	
				(false discovery rate)	Normal	Cancer
hsa-miR-375	MI0000783_1	2q35	−5.463	0.0484	14.39	8.93
hsa-miR-133a-2	MI0000451_1	20q13.33	−4.535	0.3039	9.76	5.22
hsa-miR-133a-1	MI0000450_1	18q11.2	−4.502	0.3054	9.75	5.25
hsa-miR-150-3p	MI0000479_1	19q13.33	−3.933	0.1521	5.70	1.76
hsa-miR-1-2	MI0000437_1	18q11.2	−3.644	0.3857	14.68	11.04
hsa-miR-1-1	MI0000651_1	20q13.33	−3.636	0.3864	14.72	11.08
hsa-miR-135a-2-5p	MI0000453_1	12q23.1	−3.307	0.1744	5.86	2.55
hsa-miR-135a-1-5p	MI0000452_2	3p21.1	−3.274	0.1746	5.81	2.53
hsa-miR-885-5p	MI0005560_2	3p25.3	−3.160	0.1094	4.14	0.98
hsa-miR-4521	MI0016887_1	17p13	−3.107	0.1898	9.08	5.97
hsa-miR-150-5p	MI0000479_2	19q13.33	−2.910	0.2039	13.41	10.50
hsa-miR-139-5p	MI0000261_2	11q13.4	−2.863	0.0763	12.36	9.49
hsa-miR-504	MI0003189_1	Xq26.3	−2.745	0.1818	7.43	4.69
hsa-miR-497-5p	MI0003138_2	17p13.1	−2.678	0.1496	7.89	5.21
hsa-miR-99a-3p	MI0000101_2	21q21.1	−2.446	0.0749	8.17	5.72
hsa-miR-100-5p	MI0000102_2	11q24.1	−2.438	0.1006	17.31	14.87
hsa-miR-99a-5p	MI0000101_1	21q21.1	−2.389	0.0107	18.21	15.83
hsa-miR-125b-2-5p	MI0000470_1	21q21.1	−2.373	0.0863	15.65	13.28
hsa-miR-125b-1-5p	MI0000446_2	11q24.1	−2.344	0.0882	15.64	13.29
hsa-miR-338-3p	MI0000814_1	17q25.3	−2.205	0.2557	6.28	4.07
hsa-miR-582-5p	MI0003589_2	5q12.1	−2.095	0.1636	8.05	5.95
hsa-miR-451a	MI0001729_1	17q11.2	−2.063	0.3321	14.56	12.50
hsa-miR-887	MI0005562_1	5p15.1	−2.053	0.2633	5.06	3.00
hsa-miR-1247-5p	MI0006382_2	14q32.31	−2.034	0.2610	4.61	2.58
hsa-miR-195-5p	MI0000489_2	17p13.1	−2.028	0.1470	11.16	9.13
hsa-miR-144-5p	MI0000460_2	17q11.2	−1.963	0.2622	8.49	6.52
hsa-let-7c	MI0000064_1	21q21.1	−1.931	0.0432	15.29	13.36
hsa-miR-29c-3p	MI0000735_1	1q32.2	−1.929	0.3842	10.44	8.51
hsa-miR-145-5p	MI0000461_1	5q32	−1.927	0.1849	15.26	13.34
hsa-miR-199b-5p	MI0000282_2	9q34.11	−1.896	0.2647	16.03	14.13
hsa-miR-29c-5p	MI0000735_2	1q32.2	−1.872	0.2625	8.28	6.41
hsa-miR-126-5p	MI0000471_1	9q34.3	−1.836	0.2607	12.79	10.95
hsa-miR-29a-3p	MI0000087_1	7q32.3	−1.798	0.1147	14.70	12.91
hsa-miR-664-3p	MI0006442_1	1q41	−1.781	0.1149	9.84	8.06
hsa-miR-125b-1-3p	MI0000446_1	11q24.1	−1.723	0.2313	8.69	6.96
hsa-miR-140-3p	MI0000456_2	16q22.1	−1.721	0.1487	14.08	12.36
hsa-miR-338-5p	MI0000814_2	17q25.3	−1.703	0.2685	7.45	5.75
hsa-miR-486-5p	MI0002470_2	8p11.21	−1.702	0.2652	11.98	10.27
hsa-miR-10b-5p	MI0000267_1	2q31.1	−1.682	0.0482	17.08	15.40
hsa-miR-29a-5p	MI0000087_2	7q32.3	−1.648	0.4284	4.44	2.79
hsa-miR-1468	MI0003782_1	Xq11	−1.611	0.2468	4.65	3.03
hsa-miR-10b-3p	MI0000267_2	2q31.1	−1.585	0.1037	6.49	4.91
hsa-miR-140-5p	MI0000456_1	16q22.1	−1.582	0.2556	11.22	9.64
hsa-miR-195-3p	MI0000489_1	17p13.1	−1.565	0.3212	8.58	7.02
hsa-miR-203	MI0000283_1	14q32.33	−1.564	0.3170	17.58	16.02
hsa-miR-585	MI0003592_1	5q35.1	−1.564	0.1830	6.76	5.20
hsa-miR-126-3p	MI0000471_2	9q34.3	−1.560	0.1626	17.53	15.97
hsa-miR-145-3p	MI0000461_2	5q32	−1.552	0.2569	9.56	8.01
hsa-miR-26b-5p	MI0000084_1	2q35	−1.547	0.0804	15.66	14.12
hsa-miR-29b-2-5p	MI0000107_2	1q32.2	−1.531	0.1254	6.52	4.99
hsa-miR-154-5p	MI0000480_1	14q32.31	−1.524	0.2568	5.64	4.11
hsa-miR-146a-5p	MI0000477_1	5q33.3	−1.502	0.2794	13.38	11.88

### Expression levels of *miR-150-5p* and *miR-150-3p* in HNSCC clinical specimens and cell lines

The stem-loop sequence of *miR-150* and the mature sequences of *miR-150-5p* and *miR-150-3p* are shown in Supplementary Figure 1A. Database (http://www.mirbase.org/) indicates that *miR-150-5p* is recognized as a guide strand and *miR-150-3p* as a passenger strand.

**Figure 1:**
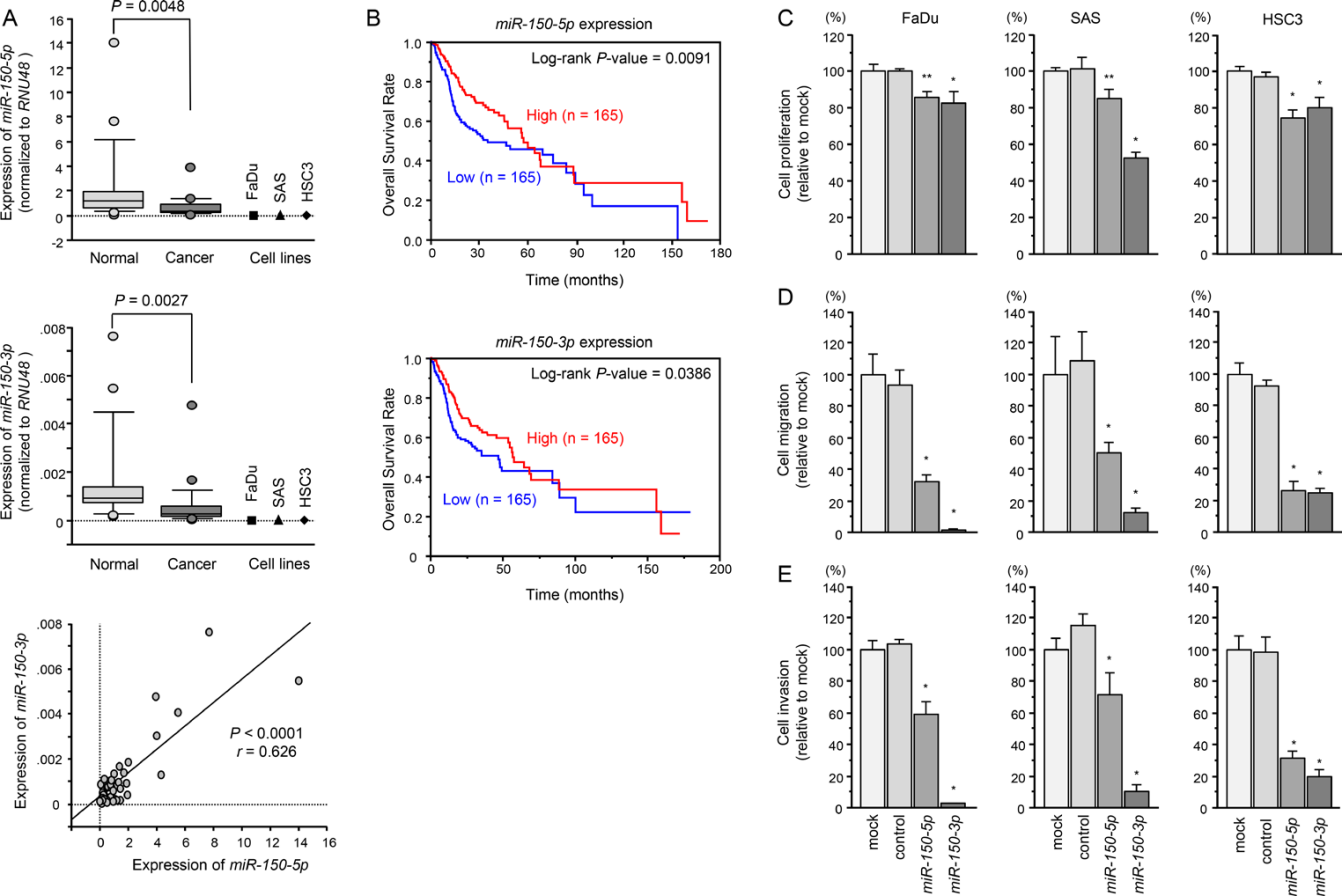
Expression levels of *miR-150-5p* and *miR-150-3p* in HNSCC clinical specimens and functional significance of *miR-150-5p* and *miR-150-3p* in HNSCC cells (**A**) Expression levels of *miR-150-5p* and *miR-150-3p* in HNSCC clinical specimens and cell lines. *RNU48* was used as an internal control. Spearman's rank test showed a positive correlation between the expressions of *miR-150-5p* and *miR-150-3p*. (**B**) Kaplan–Meier survival curves, as determined using data from the TCGA database. (**C**) Cell proliferation was determined by XTT assay 72 h after transfection with *miR-150-5p* and *miR-150-3p*. *P < 0.0001, **P < 0.0005. (**D**) Cell movement was assessed by migration assays 48 h after transfection with *miR-150-5p* and *miR-150-3p*. *P < 0.0001. (**E**) Cell invasion was characterised by invasion assays 48 h after transfection with *miR-150-5p* and *miR-150-3p*. *P < 0.0001.

Expression levels of *miR-150-5p* and *miR-150-3p* in HNSCC tissues (*n* = 22), normal epithelial tissues (*n* = 22), and three HNSCC cell lines (including FaDu, SAS and HSC3 cells) were evaluated. Clinical features of patients with HNSCC are summarised in Table 1. The expression levels of *miR-150-5p* and *miR-150-3p* were markedly lower in tumor tissues and HNSCC cell lines than in normal epithelial tissues (*P* = 0.0048 and *P* = 0.0027, respectively, Figure 1A). Spearman's rank tests showed positive correlations between the expression of *miR-150-5p* and *miR-150-3p* (R = 0.626 and *P* < 0.0001; Figure 1A).

A large cohort analysis (*n* = 330) using data from the TCGA database revealed that low expression of *miR-150-5p* and *miR-150-3p* predicted the overall survival of HNSCC patients (*P* = 0.0091 and *p* = 0.0386, respectively; Figure 1B).

### Both strands of miRNAs (*miR-150-5p* and *miR-150-3p*) incorporated into RISC in SAS cells

We hypothesized that passenger strand of *miR-150-3p* might be incorporated into the RISC and functioned in cancer cells. We performed immunoprecipitation with antibodies targeting Ago2, which plays a central role in the RISC. After transfection with *miR-150-5p* or *miR-150-3p*, Ago2-bound miRNAs were isolated, and RT-qPCR was carried out to determine whether *miR-150-5p* and *miR-150-3p* bound to Ago2 (Supplementary Figure 1B).

After transfection with *miR-150-5p* and immunoprecipitation by anti-Ago2 antibodies, detection level of *miR-150-5p* was significantly higher than those of mock- or miR-control-transfected cells and those of *miR-150-3p*-transefected SAS cells (*P* < 0.0001; Supplementary Figure 1C). Likewise *miR-150-5p* transfection, *miR-150-3p* was detected by Ago2 immunoprecipitation (*P* < 0.0001; Supplementary Figure 1C).

### Ectopic expression effects of *miR-150-5p* and *miR-150-3p* in HNSCC cell lines

The anti-tumor roles of *miR-150-5p* and *miR-150-3p* were in investigated by using mature miRNA transfection into cancer cell lines, FaDu, SAS and HSC3.

Cancer cell proliferations were inhibited by transfected with *miR-150-5p* or *miR-150-3p* in comparison with mock or control transfectants (*P < 0.0001, **P < 0.0005, respectively; Figure 1C). Migration activities were significantly suppressed after transfection with *miR-150-5p* or *miR-150-3p* (*P* < 0.0001, Figure 1D). Similarly, invasion assays showed that transfection with *miR-150-5p* or *miR-150-3p* significantly suppressed cell invasion activity (*P* < 0.0001; Figure 1E).

Synergistic effects of *miR-150-5p* and *miR-150-3p* were investigated by proliferation and migration assays with co-transfection of *miR-150-5p* and *miR-150-3p* in FaDu cells. There were no apparent synergistic effects following co-transfection with these miRNAs (Supplementary Figure 2A and 2B).

### Identification of putative target genes regulated by both *miR-150-5p* and *miR-150-3p* in HNSCC cells

**Figure 2:**
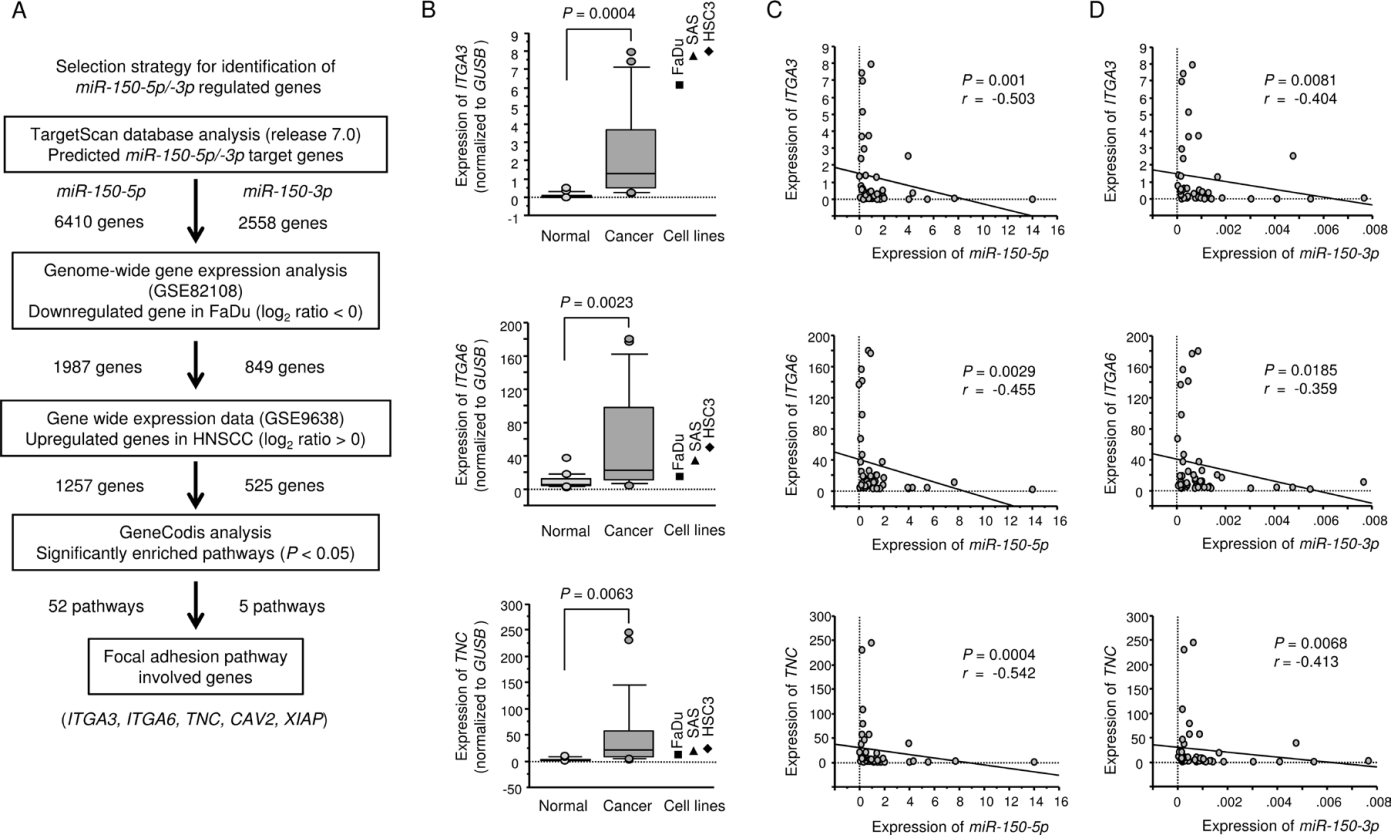
Identification of *miR-150-5p* and *miR-150-3p* target genes and expression levels of three genes in HNSCC clinical specimens (**A**) Flow chart depicting the strategy for identification of *miR-150-5p* and *miR-150-3p* target genes. (**B**) Expression levels of *ITGA3*, *ITGA6*, and *TNC* in HNSCC clinical specimens and cell lines. *GUSB* was used as an internal control. (**C**) The negative correlation between gene expression and *miR-150-5p*. Spearman's rank test was used to evaluate the correlation. (**D**) The negative correlation between gene expression and *miR-150-3p*. Spearman's rank test was used to evaluate the correlation.

To identify putative target genes coordinately regulated by *miR-150-5p* and *miR-150-3p*, we performed applied to combination of *in silico* analyses, oligomicroarray expression analyses, and Gene Omnibus database (GEO) analyses. Our strategy for selection of putative target genes that were coordinately regulated by *miR-150-5p* and *miR-150-3p* is shown in Figure 2A.

The TargetScan database (release 7.0) showed that 6,410 and 2,558 genes had putative target sites for *miR-150-5p* and *miR-150-3p*, respectively. First, we analysed genome-wide gene expression assays using FaDu cells (GEO accession number: GSE82108). A total of 1,987 and 849 genes were downregulated in *miR-150-5p* and *miR-150-3p* transfectants, respectively, and had putative binding sites in their 3′-UTRs. Next step, to evaluate upregulated genes in clinical HNSCC samples (GEO accession number: GSE9638). We identified 1,257 and 525 genes as putative target genes for *miR-150-5p* and *miR-150-3p*, respectively, in HNSCC cells.

Finally, we categorised these putative target genes into Kyoto Encyclopedia of Genes and Genomes (KEGG) pathways using the GeneCodis database, and 52 and 5 pathways were listed as putative *miR-150-5* and *miR-150-3p* regulated pathways (Tables 3 and 4). Among these pathways, we focused on the focal adhesion pathway because both *miR-150-5p* and *miR-150-3p* markedly inhibited cancer cell migration and invasion (Figure 1D and 1E). Five genes (*TNC*, *ITGA3*, *ITGA6*, *CAV2*, and *XIAP*) were found to be regulated by both *miR-150-5p* and *miR-150-3p* in this pathway (Tables 5 and 6).

**Table 3:** Significantly enriched annotations regulated by miR-150-5p

Number of genes	*P*-value	Annotations
33	7.22E-06	(KEGG) 05200: Pathways in cancer
22	3.43E-06	(KEGG) 03013: RNA transport
21	1.01E-05	(KEGG) 04141: Protein processing in endoplasmic reticulum
20	1.04E-02	(KEGG) 04010: MAPK signaling pathway
20	7.57E-06	(KEGG) 04120: Ubiquitin mediated proteolysis
20	2.30E-06	(KEGG) 04110: Cell cycle
18	3.57E-04	(KEGG) 00230: Purine metabolism
17	5.42E-04	(KEGG) 04310: Wnt signaling pathway
16	1.40E-02	(KEGG) 04510: Focal adhesion
16	1.03E-05	(KEGG) 00240: Pyrimidine metabolism
15	1.17E-03	(KEGG) 05162: Measles
14	2.82E-02	(KEGG) 05016: Huntington's disease
14	4.34E-03	(KEGG) 04910: Insulin signaling pathway
14	2.21E-03	(KEGG) 04722: Neurotrophin signaling pathway
14	4.73E-05	(KEGG) 05222: Small cell lung cancer
14	1.03E-05	(KEGG) 03008: Ribosome biogenesis in eukaryotes
14	1.03E-05	(KEGG) 05220: Chronic myeloid leukemia
13	4.22E-03	(KEGG) 03040: Spliceosome
12	1.79E-02	(KEGG) 05160: Hepatitis C
12	1.10E-03	(KEGG) 05215: Prostate cancer
11	2.57E-02	(KEGG) 05145: Toxoplasmosis
10	2.67E-02	(KEGG) 04660: T cell receptor signaling pathway
10	8.29E-03	(KEGG) 04210: Apoptosis
10	5.60E-03	(KEGG) 00564: Glycerophospholipid metabolism
10	5.60E-03	(KEGG) 03015: mRNA surveillance pathway
10	1.72E-04	(KEGG) 00510: N-Glycan biosynthesis
9	2.15E-02	(KEGG) 04012: ErbB signaling pathway
9	7.54E-03	(KEGG) 05212: Pancreatic cancer
9	7.54E-03	(KEGG) 05211: Renal cell carcinoma
9	5.90E-03	(KEGG) 04115: p53 signaling pathway
9	5.90E-03	(KEGG) 04920: Adipocytokine signaling pathway
9	5.40E-04	(KEGG) 04330: Notch signaling pathway
8	2.68E-02	(KEGG) 04662: B cell receptor signaling pathway
8	1.19E-02	(KEGG) 05214: Glioma
8	1.01E-02	(KEGG) 04623: Cytosolic DNA-sensing pathway
8	1.03E-04	(KEGG) 03020: RNA polymerase
7	1.47E-02	(KEGG) 04150: mTOR signaling pathway
7	5.77E-03	(KEGG) 03420: Nucleotide excision repair
7	5.68E-03	(KEGG) 00970: Aminoacyl-tRNA biosynthesis
6	4.91E-02	(KEGG) 05223: Non-small cell lung cancer
6	2.93E-02	(KEGG) 00561: Glycerolipid metabolism
6	1.95E-02	(KEGG) 05219: Bladder cancer
6	1.63E-02	(KEGG) 03050: Proteasome
6	9.83E-03	(KEGG) 04130: SNARE interactions in vesicular transport
6	9.83E-03	(KEGG) 03030: DNA replication
6	8.16E-03	(KEGG) 03410: Base excision repair
5	1.27E-02	(KEGG) 00534: Glycosaminoglycan biosynthesis - heparan sulfate
5	1.12E-02	(KEGG) 00563: Glycosylphosphatidylinositol(GPI)-anchor biosynthesis
4	3.60E-02	(KEGG) 04320: Dorso-ventral axis formation
4	3.12E-02	(KEGG) 00532: Glycosaminoglycan biosynthesis - chondroitin sulfate
4	1.83E-02	(KEGG) 00670: One carbon pool by folate
3	2.79E-02	(KEGG) 00740: Riboflavin metabolism

**Table 4:** Significantly enriched annotations regulated by miR-150-3p

Number of genes	*P*-value	Annotations
16	0.0024	(KEGG) 05200: Pathways in cancer
10	0.0386	(KEGG) 04510: Focal adhesion
8	0.0021	(KEGG) 04520: Adherens junction
8	0.0036	(KEGG) 05222: Small cell lung cancer
4	0.0349	(KEGG) 00020: Citrate cycle (TCA cycle)

**Table 5:** Focal adhesion pathway regulated by miR-150-5p

Gene Symbol	Gene Name	conserved	poorly conserved	GEO9638 log_2_ ratio	GSE82108 log_2_ratio
TNC	tenascin C	0	3	1.416	−0.899
ITGA6	integrin, alpha 6	0	2	1.105	−0.052
VAV2	vav 2 guanine nucleotide exchange factor	0	2	0.976	−0.169
SHC1	SHC (Src homology 2 domain containing) transforming protein 1	0	1	0.933	−0.804
VEGFA	vascular endothelial growth factor A	0	1	0.653	−0.265
GSK3B	glycogen synthase kinase 3 beta	1	0	0.606	−0.089
ITGA3	integrin, alpha 3 (antigen CD49C, alpha 3 subunit of VLA-3 receptor)	0	9	0.583	−0.058
AKT2	v-akt murine thymoma viral oncogene homolog 2	0	2	0.533	−0.308
CAV2	caveolin 2	0	1	0.443	−0.914
ROCK1	Rho-associated, coiled-coil containing protein kinase 1	1	0	0.441	−0.226
GRB2	growth factor receptor-bound protein 2	0	2	0.382	−0.148
XIAP	X-linked inhibitor of apoptosis	0	3	0.341	−0.456
TNR	tenascin R (restrictin, janusin)	0	2	0.263	−0.285
TLN1	talin 1	0	3	0.165	−0.190
PIK3CB	phosphoinositide-3-kinase, catalytic, beta polypeptide	1	0	0.092	−0.068
CRK	v-crk sarcoma virus CT10 oncogene homolog (avian)	0	1	0.001	−0.069

**Table 6:** Focal adhesion pathway regulated by miR-150-3p

Gene Symbol	Gene Name	poorly conserved	GSE9638 log_2_ ratio	GSE82108 log_2_ ratio
LAMC2	laminin, gamma 2	1	2.334	−0.8519
EGFR	epidermal growth factor receptor	1	1.835	−0.6698
TNC	tenascin C	1	1.416	−0.7192
ITGA6	integrin, alpha 6	1	1.105	−0.1906
IGF1R	insulin-like growth factor 1 receptor	1	0.611	−0.0533
PTK2	PTK2 protein tyrosine kinase 2	1	0.605	−0.4804
ITGA3	integrin, alpha 3 (antigen CD49C, alpha 3 subunit of VLA-3 receptor)	1	0.583	−0.2200
CAV2	caveolin 2	1	0.443	−0.6686
XIAP	X-linked inhibitor of apoptosis	1	0.341	−0.0765
THBS1	thrombospondin 1	2	0.189	−0.1310

**Figure 3:**
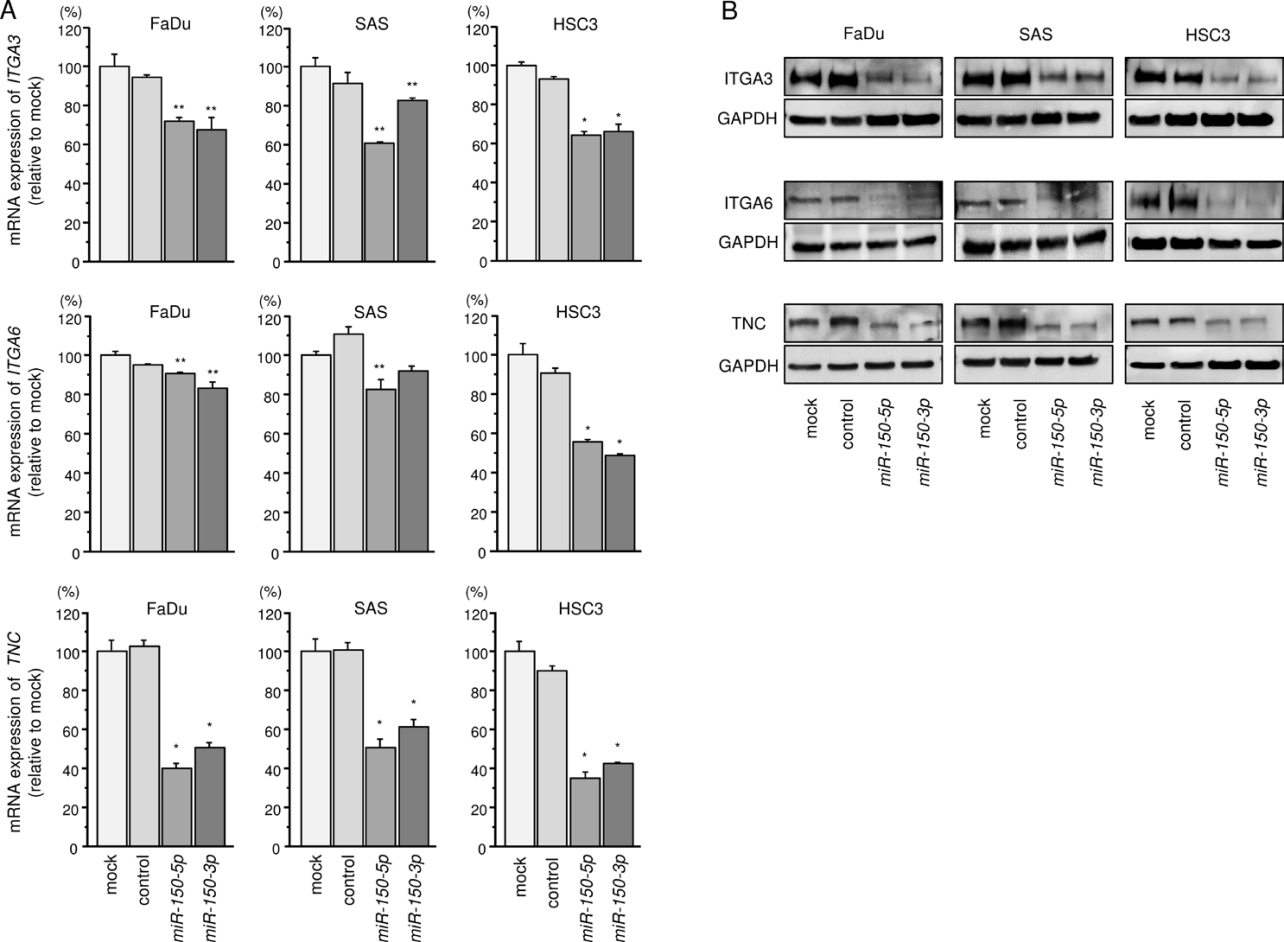
Regulation of target gene expression by *miR-150-5p* and *miR-150-3p* in HNSCC cell lines (**A**) Expression levels of *ITGA3*, *ITGA6*, and *TNC* mRNAs 72 h after transfection with 10 nM *miR-150-5p* or *miR-150-3p* into cell lines. *GUSB* was used as an internal control. *P < 0.0001, **P < 0.008. (**B**) Protein expression of ITGA3, ITGA6, and TNC 72 h after transfection with *miR-150-5p* or *miR-150-3p*. GAPDH was used as a loading control.

We next performed qRT-PCR analyses of 3 HNSCC cell lines to investigate whether restoration of *miR-150-5p* or *miR-150-3p* expression altered the mRNA expression of these 5 genes. The mRNA expression levels of these 5 candidate genes are shown in Figure 3A and Supplementary Figure 3A. Among 5 genes, we focused on *ITGA3*, *ITGA6*, and *TNC* genes because aberrant integrin-mediated signalling promoted cancer cell aggressiveness according to our previous studies [21, 22, 26]. Moreover, two genes *CAV* and *XIAP* have been involved in cancer pathogenesis including HNSCC by past studies [27–30]. The analysis of the expression control of these two genes by miRNAs is important theme.

### Regulation of *ITGA*3, *ITGA6*, and *TNC* expression by *miR-150-5p* and *miR-150-3p* in HNSCC clinical specimens and HNSCC cells

Next, we investigated the mRNA expression levels of *ITGA3*, *ITGA6*, and *TNC* in 22 HNSCC clinical specimens by qRT-PCR. *ITGA3*, *ITGA6*, and *TNC* were significantly upregulated in HNSCC tissues (*P* = 0.0004, *P* = 0.0023, and *P* = 0.0063, respectively; Figure 2B). Spearman's rank tests showed a negative correlation between the expression levels of these 3 genes and *miR-150-5p* (*P* = 0.001, *P* = 0.0029, and *P* = 0.0004, respectively; R = −0.503, R = −0.455, and R = −0.542, respectively; Figure 2C) and between the expression of these 3 genes and *miR-150-3p* (*P* = 0.0081, *P* = 0.0185, and *P* = 0.0068, respectively; R = −0.404, R = -0.359, and R = −0.413, respectively; Figure 2D).

Next, we investigated whether *ITGA3*, *ITGA6*, and *TNC* expression was reduced by restoration of *miR-150-5p* and *miR-150-3p* in HNSCC cells. Expression levels of these 3 genes were markedly repressed in *miR-150-5p* and *miR-150-3p* transfectant cells compared with that in mock-transfected cells (Figure 3A and 3B).

The synergistic effects of *miR-150-5p* and *miR-150-3p* were evaluated the mRNA expression levels of *ITGA3*, *ITGA6*, and *TNC* with co-transfection of *miR-150-5p* and *miR-150-3p* in FaDu cells. However, no synergistic effects were observed (Supplementary Figure 3B).

### Immunohistochemical detection of ITGA3, ITGA6 and TNC in HNSCC clinical specimens

**Figure 4:**
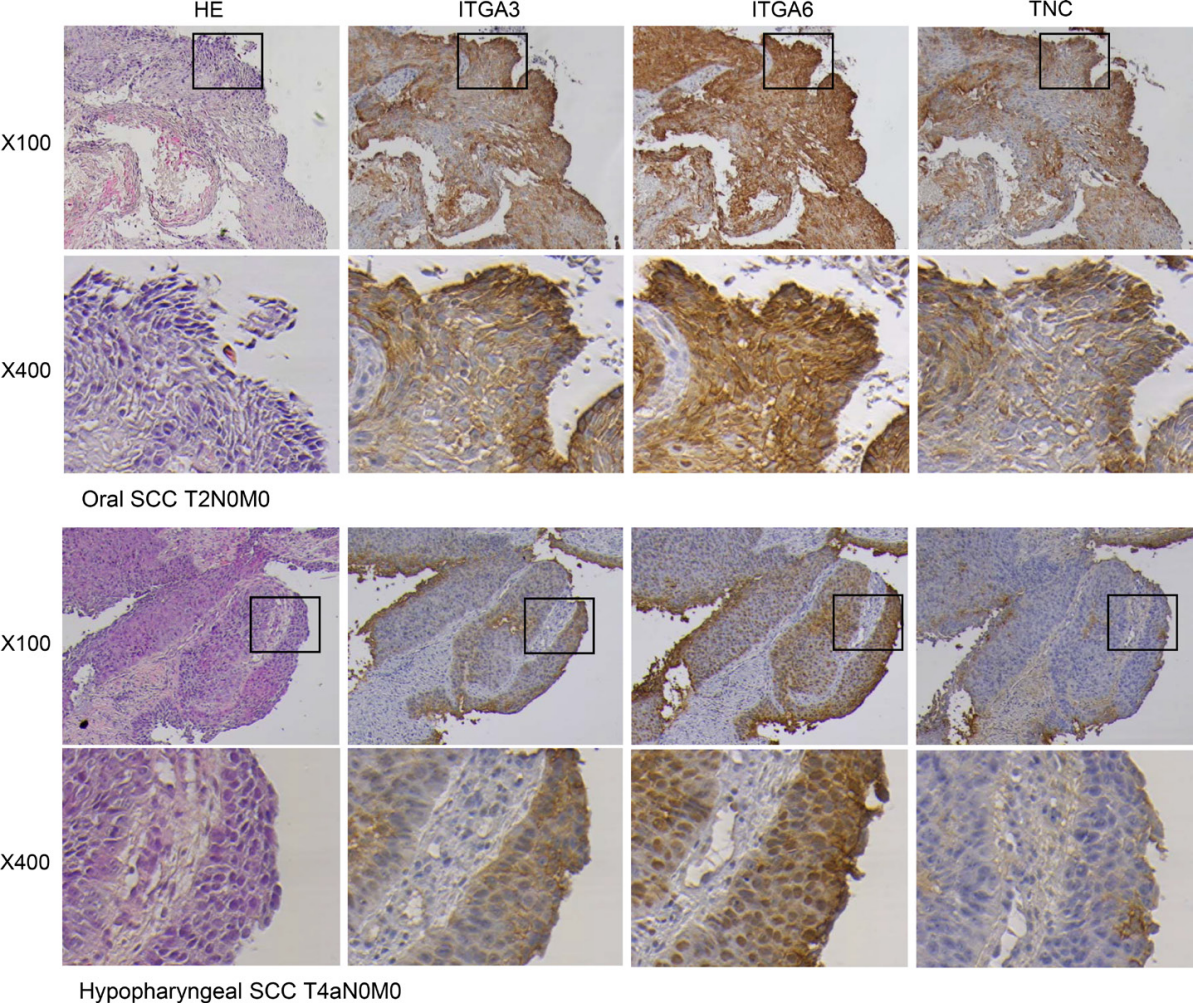
Immunohistochemical staining of ITGA3, ITGA6 and TNC in clinical specimens of HNSCC ITGA3 and ITGA6 were strongly expressed in cancer lesions, while TNC was moderately expressed in cancer lesions. (100× and 400× magnification field).

We also examined the expression levels of ITGA3, ITGA6 and TNC in HNSCC clinical specimens by immunohistochemical staining. ITGA3 and ITGA6 were strongly expressed in several cancer tissues (Figure 4). TNC was moderately expressed in cancer lesions (Figure 4). While low expression was observed in noncancerous regions (Figure 4).

### Effects of *ITGA3*, *ITGA6*, and *TNC* knockdown on cell proliferation, migration, and invasion in FaDu cells

**Figure 5:**
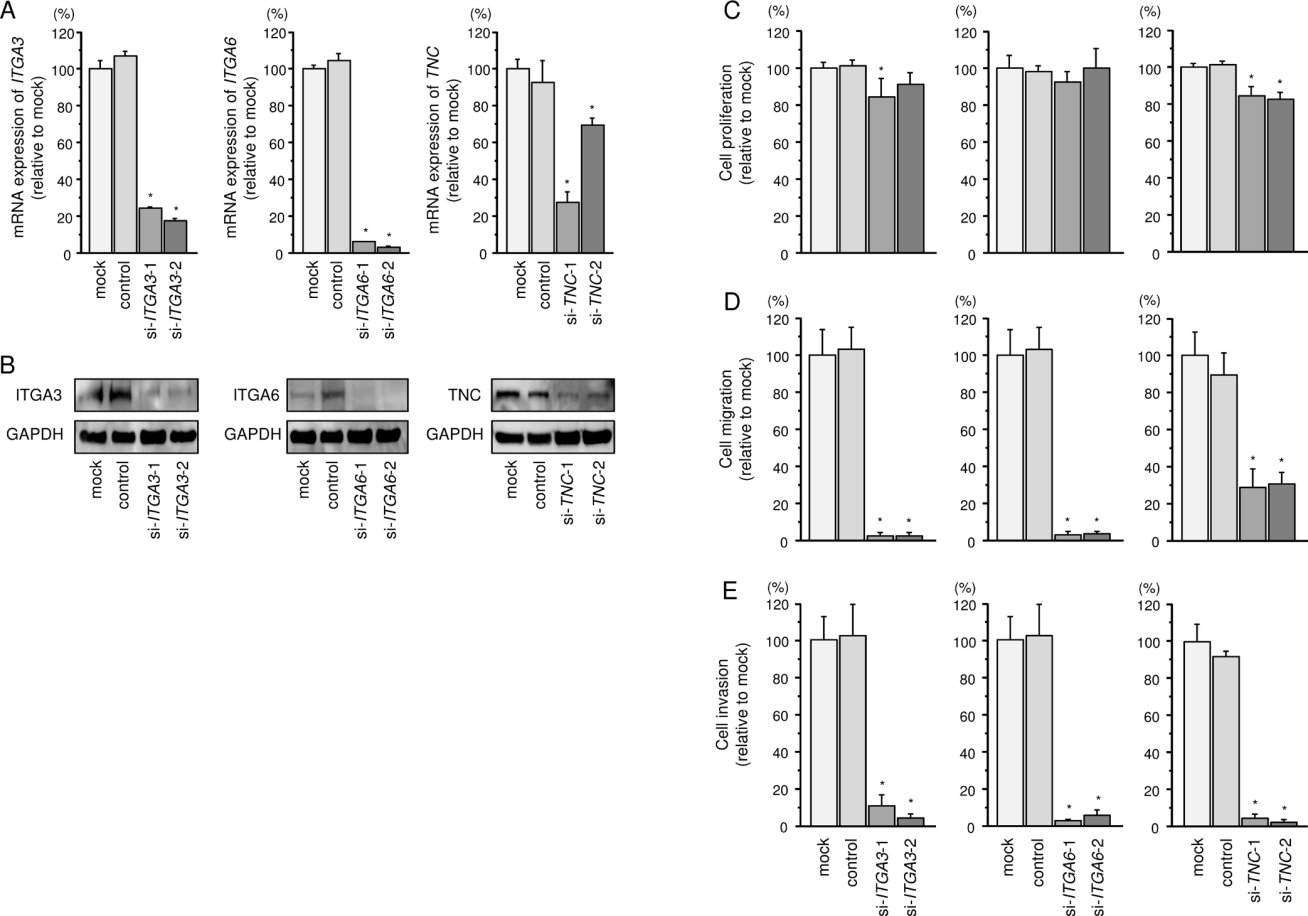
Effects of *ITGA3*, *ITGA6*, and *TNC* silencing by siRNA transfection in FaDu cells (**A**) *ITGA3*, *ITGA6*, and *TNC* mRNA expression 72 h after transfection with 10 nM siRNA into FaDu cells. *GUSB* was used as an internal control.*P < 0.0001. (**B**) ITGA3, ITGA6, and TNC protein expression 72 h after transfection with siRNA. GAPDH was used as a loading control. (**C**) Cell proliferation was determined by XTT assay 72 h after transfection with siRNA. *P < 0.001. (**D**) Cell movement was assessed by migration assay 48 h after transfection with siRNA. *P < 0.0001. (**E**) Cell invasion was characterised by invasion assay 48 h after transfection with siRNA. *P < 0.0001.

To investigate the oncogenic functional of *ITGA3*, *ITGA6*, and *TNC* in FaDu cells, we applied to loss-of-function assays using siRNAs. First, we evaluated the knockdown efficiency of si-*ITGA3*, si-*ITGA6*, and si-*TNC* transfection in FaDu cells. Knockdown efficiencies of siRNAs, si-*ITGA3*, si-*ITGA6*, and si-*TNC* were shown in Figure 5A and 5B.

Cancer cells proliferations were significantly reduced in *si-ITGA3-1* and *si-TNC* transfectants in comparison with that in mock-transfected FaDu cells (Figure 5C). In contrast, proliferation was not inhibited in FaDu cells transfected with *si-ITGA3-2* and *si-ITGA6*. Migration activities were significantly suppressed in *si-ITGA3*, *si-ITGA6*, and *si-TNC* transfectants in comparison with that in mock-transfected FaDu cells (Figure 5D). As like as migration assays, invasion activity was significantly inhibited in *si-ITGA3*, *si-ITGA6*, and *si-TNC* transfectants in comparison with that in mock-transfected FaDu cells (Figure 5E).

### Clinical significance of *ITGA3*, *ITGA6*, and *TNC* expression in HNSCC

**Figure 6:**
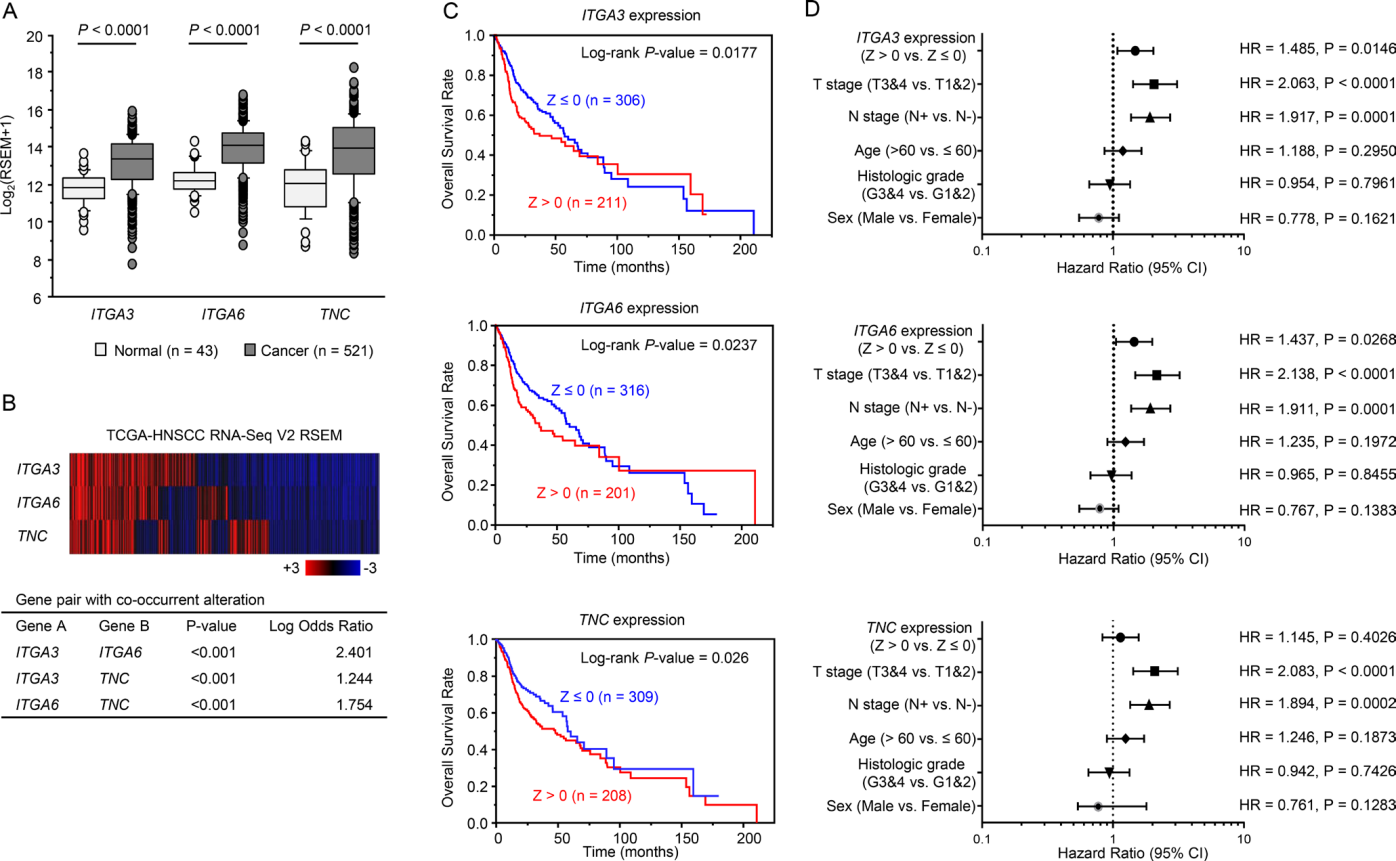
Clinical significance of three genes and *miR-150-5p* and *miR-150-3p* expression in HNSCC based on data from the TCGA database (**A**) Comparison of *ITGA3*, *ITGA6*, and *TNC* mRNA expression levels between normal and tumor samples in TCGA database. (**B**) Heatmap visualization of gene expression based on TCGA-HNSCC RNA Seq data (Upper). All gene pairs among three genes expression pattern (mRNA Z-score > 0) showed significant co-occurrence by Fisher's exact test (Lower). (**C**) Clinical outcomes in patients with altered *miR-150-5p* and *miR-150-3p* expression (CNV: amplification or gain, or mRNA: Z-score > 0) or without altered miRNA expression (CNV: diploid or het loss, and mRNA: Z-score ≤ 0), as displayed using Kaplan–Meier plots with log-rank tests. (**D**) Forest plot summarising hazard ratios. The X-axis displays the hazard ratio and 95% CI of each subgroup.

To investigate the clinical significance of *ITGA3*, *ITGA6*, and *TNC* in HNSCC, we analysed their associations with tumor stage and lymph node stage using the TCGA-PRAD database. The mRNA expression levels of *ITGA3*, *ITGA6*, and *TNC* were significantly upregulated in HNSCC clinical samples (Figure 6A).

Heatmap visualization of gene expression showed all gene pairs among three genes expression pattern (mRNA Z-score > 0) show significant co-occurrence (Figure 6B).

**Figure 7:**
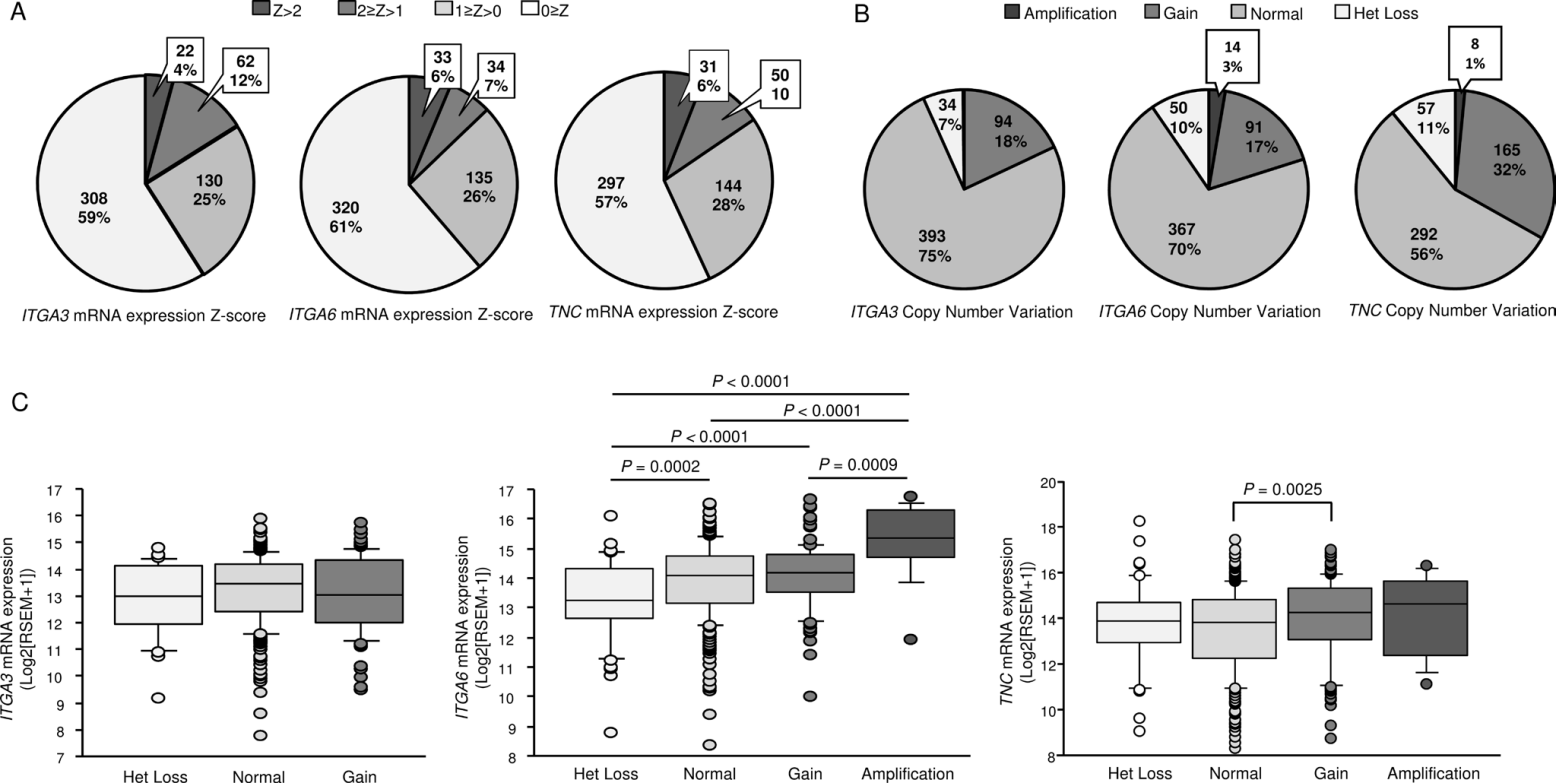
(**A**, **B**) Distributions of *ITGA3*, *ITGA6*, and *TNC* mRNA Z-scores (*n* = 522) and genomic copy number variations (*n* = 522) in HNSCC, as determined using data from TCGA. (**C**) Box-and-whisker plots of *ITGA3*, *ITGA6*, and *TNC* mRNA expression with respect to genomic copy number.

Next, to examine whether the expression levels of these genes predicted overall survival, patients were divided into two groups: Z-score > 0 and Z-score ≤ 0. Higher *ITGA3*, *ITGA6*, and *TNC* expression levels were associated with shorter overall survival (*P* = 0.0177, *P* = 0.0237, *P* = 0.026, respectively; Figure 6C). Details of mRNA expression z-scores are described in Figure 7A. The distributions of *ITGA3*, *ITGA6*, and *TNC* genomic copy number variations are shown in Figure 7B and 7C.

Multivariate Cox proportional hazards models were used to assess independent predictors of progression-free survival, including expression level of the gene (Z score > 0 versus Z score ≤ 0), tumor stage (T3 and T4 versus T1 and T2), lymph node stage (N+ versus N-), age at diagnosis (> 60 years versus ≤ 60 years), histologic grade (G3 and G4 versus G1 and G2), and sex (male versus female). High *ITGA3* expression was a significant prognostic factor in patients with HNSCC (hazard ratio [HR] = 1.485, 95% confidence interval [CI] = 1.082–2.035, *P* = 0.0146; Figure 6D). Likewise, high *ITGA6* expression (HR = 1.437, 95% CI = 1.043–1.975, *P* = 0.0268) was a significant prognostic factor (Figure 6D). Furthermore, the expression of *ITGA3*, *ITGA6* and *TNC* genes were significantly higher in the T3 and T4 group than in the T1 and T2 group (HR = 2.063, HR = 2.133, *HR* = 2.083, respectively; *P* < 0.0001; Figure 6D).

**Figure 8:**
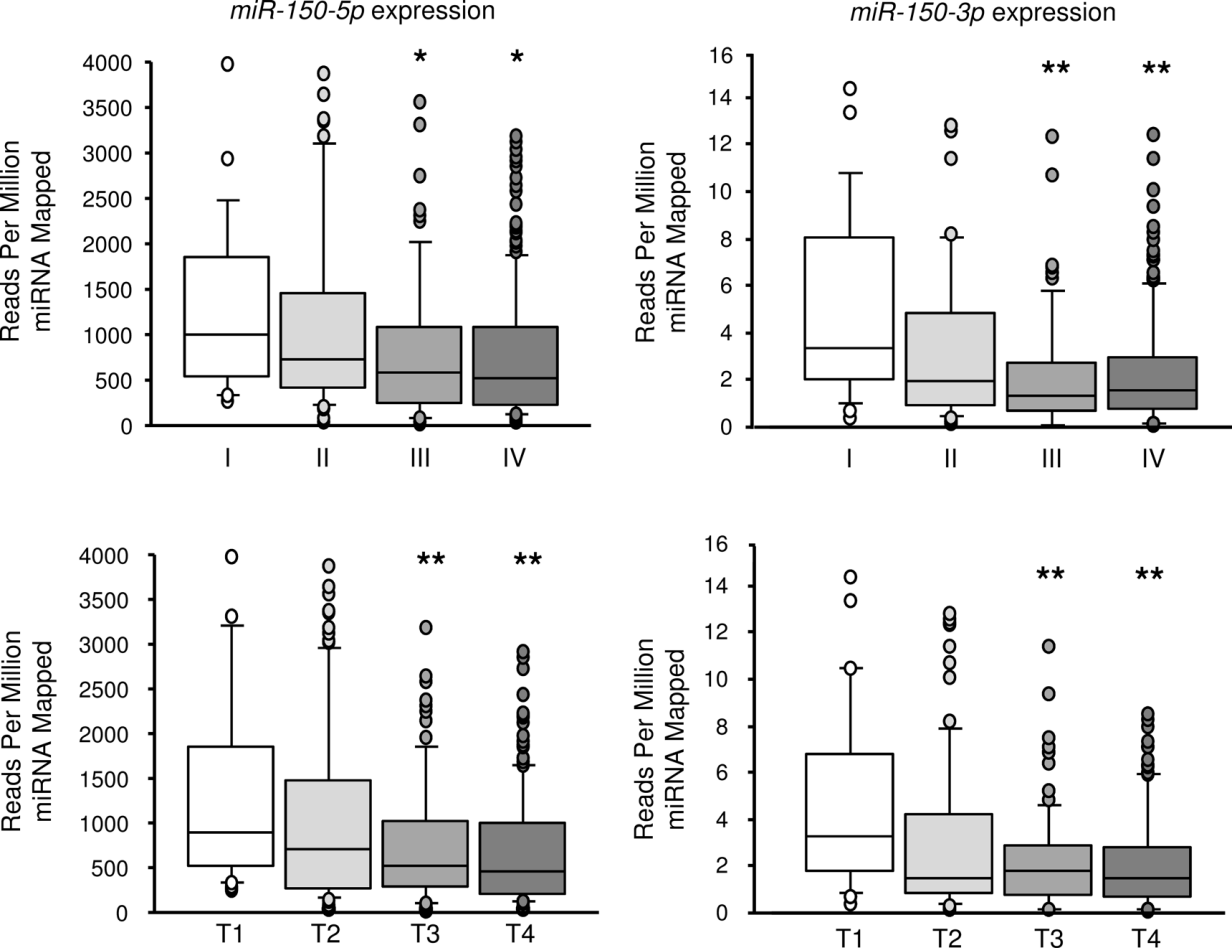
Expression of *miR-150-5p* and *miR-150-3p* according to TNM stage and T stage (compared with T1 or stage I) From the data of the TCGA database, the expression level of *miR-150-5p* and *miR-150-3p* was significantly decreased in advanced cases and advanced T stage cases. *P < 0.05, **P < 0.01.

From the data of the TCGA database, the expression level of *miR-150-5p* and *miR-150-3p* were extracted for each TNM stage and T stage. The expression level of *miR-150-5p* and *miR-150-3p* was significantly decreased in advanced cases. The expression level of *miR-150-5p* and *miR-150-3p* were significantly decreased in advanced T stage cases (Figure 8).

## DISCUSSION

The control of intercellular protein-coding/noncoding RNA expression is tightly regulated by miRNA [6]. Therefore, dysregulation of miRNAs can profoundly impact normal cell physiology and enhance oncogenesis. Identification of oncogenic and antitumor miRNAs has led us to elucidate novel miRNA-mediated cancer networks. We have sequentially obtained the miRNA expression signatures of several types of cancers and used these signatures to identify antitumor miRNA-mediated cancer pathways [11–19, 31]. Deep sequencing technology can be used to illuminate the miRNA expression signatures of cancer cells. In this study, we created deep sequencing-based miRNA expression signatures using laryngeal and hypopharyngeal squamous cell carcinoma clinical specimens.

Our signature included several antitumor miRNAs (*miR-375*, *miR-133a*, and *miR-1* etc.) that had been identified in human cancers in previous studies [32–40], suggesting that our present signature was effective for identification of novel oncogenic and antitumor miRNAs in HNSCC cells and may provide a benchmark for future studies of HNSCC. Interestingly, our present signature revealed that both strands of *pre-miR-150*, i.e., *miR-150-5p* and *miR-150-3p* were significantly downregulated in this signature. Our recent studies demonstrated that both strands of *pre-miR-144* (*miR-144-5p*: guide strand and *miR-144-3p*: passenger strand) and *pre-miR-145* (*miR-145-5p*: guide strand and *miR-145-3p*: passenger strand) inhibited cancer cell aggressiveness [41–43]. These findings suggested that passenger strand of miRNAs had antitumor function in several cancer cells. Therefore, we focused on *miR-150-5p* and *miR-150-3p* and investigated the antitumor functional of these miRNAs in HNSCC cells.

Our present data demonstrated that both *miR-150-5p* and *miR-150-3p* were significantly reduced in cancer tissues, and ectopic expression of each miRNA markedly inhibited cancer cell aggressiveness. These data indicated that both strands of *miR-150-5p* and *miR-150-3p* functioned as antitumor miRNAs in HNSCC cells. Past studies have reported the aberrant expression of *miR-150-5p* in various cancers, suggesting that this miRNA contributes significantly to cancer pathogenesis [44]. However, the expression status of *miR-150-5p* was varied according to cancer type, indicating that *miR-150-5p* was a multifunction molecule with both oncogenic and antitumor functions depending on the cancer type [44]. In contrast, the functional significance of *miR-150-3p* in human cancer is still unclear. Our present study is the first report demonstrating that *miR-150-3p* acts as an antitumor miRNA, similar to *miR-150-5p*, in HNSCC cells.

Molecular mechanism of silencing of *miR-150-5p* and *miR-150-3p* in HNSCC cells is still obscure. To investigate these miRNAs expression and DNA methylation, SAS cells were treated with the demethylating agent (5-aza-dC). Expression of *miR-150-5p* was significantly elevated after 5-aza-dC treatment (Supplementary Figure 4). On the other hand, expression of *miR-150-3p* was slightly upregulated by 5-aza-dC treatment (Supplementary Figure 4). These data suggested that DNA methylation might be contributed to silencing of *miR-150-5p* and *miR-150-3p* in HNSCC cells.

We also evaluated the roles of *miR-150-5p* and *miR-150-3p* in regulating genes and pathways in HNSCC cells. We hypothesised that *miR-150-5p* and *miR-150-3p* may coordinately regulate target genes associated with HNSCC pathogenesis. To identify miRNA-regulated targets and pathways, we applied *in silico* and gene expression analyses, as described in our previous studies [13–17, 41]. Here, we identified 5 putative candidate genes involved in the focal adhesion pathway (*TNC*, *ITGA3*, *ITGA6*, *CAV2*, and *XIAP*) that were regulated by both *miR-150-5p* and *miR-150-3p* in HNSCC cells. Among these genes, our previous studies demonstrated that *ITGA3*, *ITGA6*, and *CAV2* were overexpressed in cancer tissues and associated with cancer cell migration and invasion [22, 26, 28]. Moreover, *ITGA3*, *ITGA6*, and *CAV2* have been shown to be directly regulated by antitumor miRNAs, i.e., *miR-223*, *miR-29s*, and *miR-218*, respectively [22, 26, 28]. In this study, we focused on *TNC* and investigated its functional roles in HNSCC.

*TNC* is a multifunctional extracellular matrix (ECM) glycoprotein composed of several distinct domains [45]. Expression of *TNC* in adult tissue is restricted, except in the context of inflammation and tissue injury [45]. Cancer cells and corresponding stromal cells also exhibit *TNC* expression, and high expression of *TNC* has been reported in several cancers [45–47]. Overexpression of *TNC* induces ECM deposition, and aberrant activation of ECM-mediated signalling promotes cancer cell aggressiveness, and the epithelial-mesenchymal transition (EMT) [45–47]. Several studies have demonstrated that *TNC* binds directly to integrins, and TNC/integrin-mediated signalling contributes to embryonic development, tissue repair, and cancer pathogenesis [46, 48]. Our previous studies showed that overexpression of *ITGA6*/*B4* and *ITGA3*/*B1* was involved in cancer cell migration and invasion in HNSCC and prostate cancer [22, 26]. Moreover, a recent study showed that *ITGA6* is a hypoxia-inducible factor (HIF)-dependent transcriptional target gene and that *ITGA6* expression is an independent prognostic factor in patients with breast cancer [49].

Our large cohort study using TCGA datasets indicated that the expression levels of *ITGA3*, *ITGA6*, and *TNC* were upregulated in cancer tissues. Furthermore, Kaplan–Meier survival curves revealed that high expression of these genes predicted poorer survival in patients with HNSCC. Our functional study showed that silencing of *ITGA3*, *ITGA6*, and *TNC* inhibited HNSCC cell migration and invasion. Metastasis is responsible for most of the mortality in patients with HNSCC. Therefore, activation of TNC/integrin receptor-mediated signalling may be a putative target in cancer treatment.

In conclusion, downregulation of dual strands of pre-*miR-150* (*miR-150-5p* and *miR-150-3p*) was detected by deep sequencing-based miRNA signature analysis. The antitumor functions of these miRNAs coordinately regulate focal adhesion pathway-related genes in HNSCC cells. The expression of *ITGA3*, *ITGA6*, and *TNC* is involved in HNSCC pathogenesis. Identification of novel cancer networks mediated by aberrantly expressed miRNAs and antitumor miRNAs may improve our understanding of HNSCC molecular pathogenesis. Our newly created deep sequencing-based miRNA signature provides a basis for further HNSCC research.

## MATERIALS AND METHODS

### Clinical HNSCC specimens, cell lines, and RNA extraction

A total of 22 clinical tissue specimens were collected from patients with HNSCC who underwent surgical resection at Chiba University Hospital between 2008 and 2014. Clinicopathological features of patients with HNSCC are summarised in Table 1. All patients in this study provided informed consent, and the study protocol was approved by the Institutional Review Board of Chiba University.

Three human HNSCC cell lines, i.e., FaDu, SAS and HSC3, were investigated in this study. All cell lines were obtained from RIKEN Cell Bank (Tsukuba, Ibaraki, Japan).

Total RNA, including miRNA, was isolated using TRIzol reagent (Invitrogen, Carlsbad, CA, USA).

### Small RNA deep sequencing and data mining

To obtain the miRNA expression signature, we carried out high-throughput deep sequencing using Genome Analyzer IIx (Illumina, CA, USA) with 6 pairs of tumor and normal samples (Table 1). The procedures of small RNA sequencing and data mining were performed as described in our previous studies [16].

### Quantitative real-time reverse transcription PCR (qRT-PCR)

PCR quantification was carried out essentially as previously described [14, 32, 38, 50]. To quantify the expression level of miRNAs, we utilised stem-loop qRT-PCR for *miR-150-5p* (assay ID: 000473; Applied Biosystems, Foster City, CA, USA) and *miR-150-3p* (assay ID: 002637; Applied Biosystems) following the manufacturer's protocol. TaqMan probes and primers for *ITGA3* (Hs01076873_m1; Applied Biosystems), *ITGA6* (Hs01041011_m1), *TNC* (assay ID: Hs01115665_m1), *CAV2* (Hs00184597_m1), and *XIAP* (Hs00745222_s1) were assay-on-demand gene expression products. mRNA and miRNA data were normalised to human *GUSB* (assay ID: Hs99999908_m1; Applied Biosystems) and *RNU48* (assay ID: 001006; Applied Biosystems), respectively.

### Transfection of miRNA mimic and small interfering RNA (siRNA) into HNSCC cells

HNSCC cells were transfected with miRNA mimics for gain-of-function experiments and siRNAs for loss-of-function experiments. Pre-miR miRNA precursors (*miR-150-5p*, P/N: PM10070; *miR-150-3p*, P/N: PM12324; and negative control miR, P/N: AM17111; Applied Biosystems) were used in these assays. The following siRNAs were used in this study: stealth select RNAi siRNA, si*ITGA3* (P/N: HSS105531 and HSS179967; Invitrogen), si*ITGA6* (P/N: HSS179958 and HSS179959), and si*TNC* (P/N: HSS105145 and HSS105147). For transfection, RNAs were incubated with OPTI-MEM (Invitrogen) and Lipofectamine RNAiMax reagent (Invitrogen), as previously described [14, 32, 38, 50].

### Cell proliferation, migration, and invasion assays

Cell proliferation, migration and invasion assays were described previously [14, 32, 38, 50].

### Incorporation of *miR-150-5p* or *miR-150-3p* into RISC by Ago2 immunoprecipitation

SAS cells were transfected with 10nM miRNA by Reverse transfection. After 48h, immunoprecipitation was performed using a microRNA isolation kit, Human Ago2 (Wako, Osaka, Japan) according to the manufacturer's protocol. Expression levels of *miR-150-5p* or *miR-150-3p* were measured by RT-qPCR methods. Detection of miRNA data were normalized to the expression of *miR-26a* (assay ID: 000404; Applied Biosystems), which was not affected by *miR-150-5p* and *miR-150-3p*.

### Identification of putative genes regulated by *miR-150-5p* and *miR-150-3p* in HNSCC cells

Specific genes regulated by *miR-150-5p* and *miR-150-3p* were identified by a combination of *in silico* and genome-wide gene expression analyses. Genes regulated by *miR-150-5p* and *miR-150-3p* were listed using the TargetScan database. Oligo microarray (Agilent Technologies; Human GE 60K) was used for gene expression analysis. The microarray data were deposited into GEO (http://www.ncbi.nlm.nih.gov/geo/), with accession number GSE82108. Upregulated genes in HNSCC were obtained from publicly available data sets in GEO (accession number: GSE9638). To identify signalling pathways regulated *in silico*, gene expression data were analysed using the KEGG pathway categories with the GeneCodis program.

### Immunohistochemistry

The formalin-fixed paraffin-embedded (FFPE) tissues were used. The patients’ backgrounds and clinicopathological characteristics are summarized in Table 1. Tissue sections were incubated overnight at 4°C with anti-ITGA3 antibodies diluted 1:100 (HPA008572; Sigma-Aldrich), anti-ITGA6 antibodies diluted 1:100 (HPA012696; Sigma-Aldrich) and anti-ITGA3 antibodies diluted 1:50 (#SC-20932; Santa Cruz Biotechnology). The procedure for immunohistochemistry was described previously [26, 32].

### Western blotting

Immunoblotting was performed with rabbit anti-ITGA3 antibodies (1:250 dilution, HPA008572; Sigma-Aldrich, St. Louis, MO, USA), anti-ITGA6 antibodies (1:500 dilution, HPA012696; Sigma-Aldrich), and anti-TNC antibodies (1:400 dilution, #sc-20932; Santa Cruz Biotechnology, Dallas, TX, USA); anti-glyceraldehyde-3-phosphate dehydrogenase (GAPDH) antibodies (1:1000 dilution, ab8245; Abcam, Cambridge, UK) were used as an internal control. The procedures were described in our previous studies [14, 32, 38, 50].

### The cancer genome atlas (TCGA) database analysis of HNSCC specimens

The clinical significance of *ITGA3*, *ITGA6*, and *TNC* in HNSCC was assessed by RNA sequencing and by using a putative copy number variation (CNV) database (predicted by the GISTIC algorithm) in HNSCC-TCGA (http://cancergenome.nih.gov). The genomic and clinical data were retrieved from cBioportal (http://www.cbioportal.org/) [51] or UCSC Cancer Browser (https://genome-cancer.ucsc.edu/proj/site/hgHeatmap/) [52], which were downloaded on June 17, 2016. Specimens with alterations in *ITGA3, ITGA6*, and *TNC* and specimens without alterations were analysed by Kaplan–Meier survival curves and log-rank statistics. Heatmap of gene expression was generated by cBioportal. The *P*-values and log odds ratio of co-occurrence are determined by Fisher's exact test. Detail information on the method is described in previous paper [51].

### Statistical analysis

Relationships between expression values in 2 conditions or variables were analysed using the Mann-Whitney *U* test or Bonferroni-adjusted Mann-Whitney *U* test. Spearman's rank test was used to evaluate the correlations between the expression of *miR-150-5p* or *miR-150-3p* and target genes. We used Expert StatView software (version 5.0 SAS Institute Inc., Cary, NC, USA) for these analyses.

## SUPPLEMENTARY MATERIALS FIGURES AND TABLES




