# Supplementary material for: Deep sequencing-based microRNA expression signatures in head and neck squamous cell carcinoma: dual strands of pre-miR-150 as antitumor miRNAs
Source: Oncotarget. 2017 Mar 17;8(18):30288–304. doi: 10.18632/oncotarget.16327 (PMC5444743; doi:10.18632/oncotarget.16327)
Supplement: Supplementary file 1 [file oncotarget-08-30288-s001.pdf]

Deep sequencing-based microRNA expression signatures in head and neck squamous cell carcinoma: dual strands of pre-*miR-150* as antitumor miRNAs

Supplementary Materials

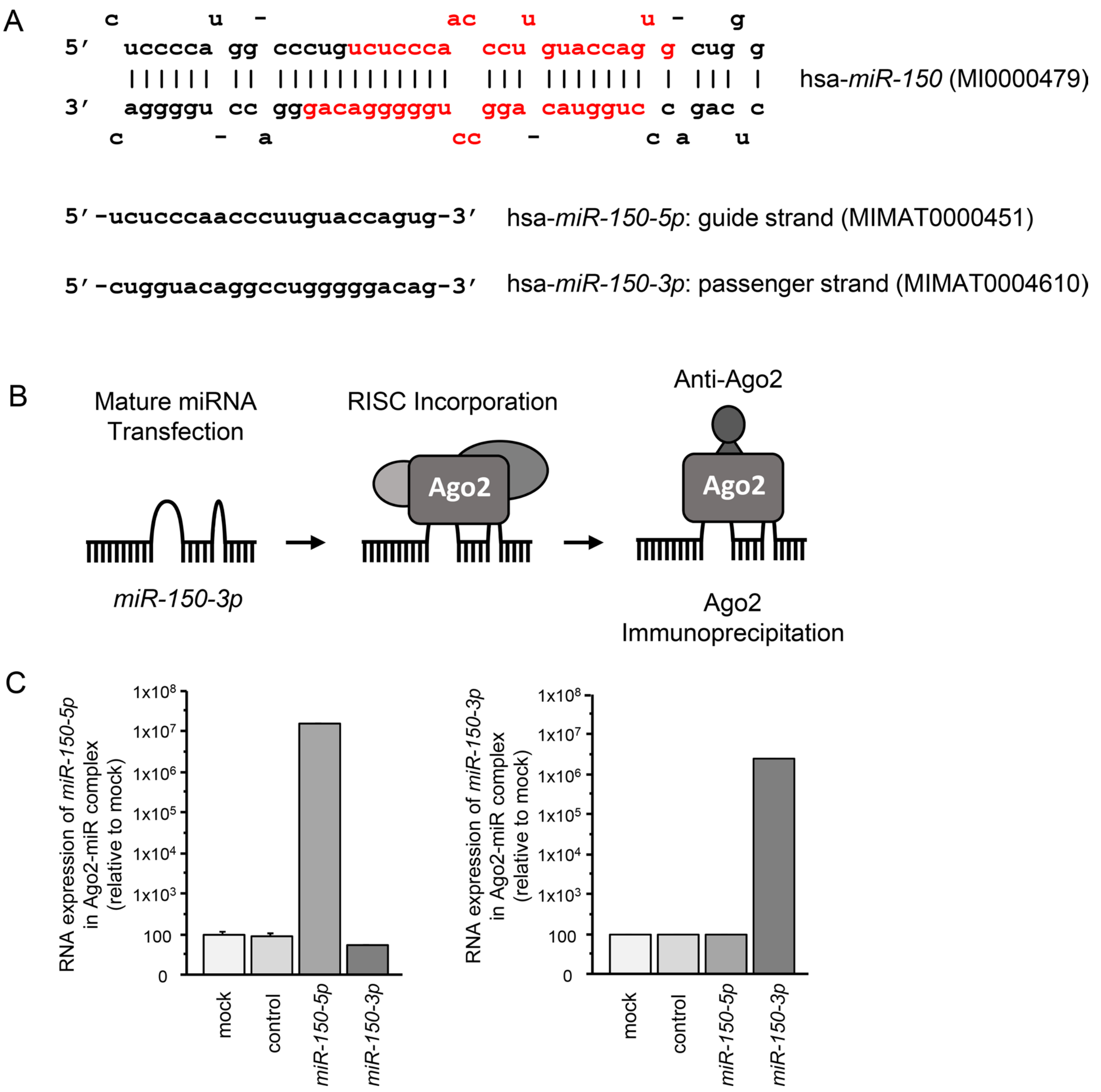

Supplementary Figure 1: Both strands of *miR-150-5p* and *miR-150-3p* incorporated into RISC. (**A**) Stem-loop sequence of *miR-150* with red characters indicating mature miRNAs was shown. Mature miRNA sequences of *miR-150-5p* and *miR-150-3p* were shown, *miR-150-5p* was recognized as guide strand, whether *miR-150-3p* as passenger strand by miRBase (http://www.mirbase.org/). (**B**) Schematic illustration of miRNA detection method. Isolation of RISC incorporated miRNAs by Ago2 immunoprecipitation. (**C**) Expression levels of *miR-150-5p* and *miR-150-3p* after transfection with miR-150-5p or *miR-150-3p* following immunoprecipitation by Ago2 ($P < 0.0001$).

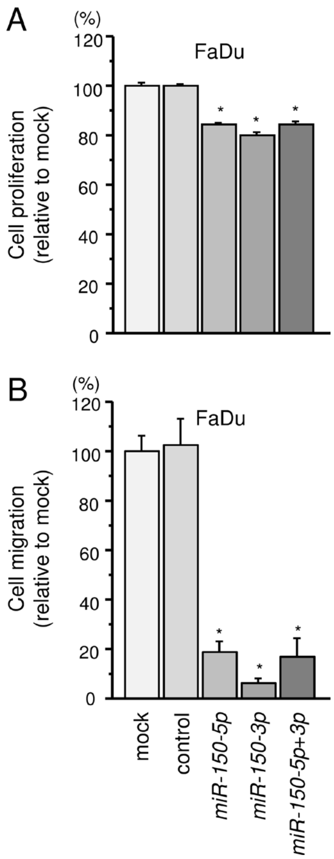

Supplementary Figure 2: The synergistic effects of *miR-150-5p* and *miR-150-3p* on HNSCC cells. (**A**) Cell proliferation was determined by XTT assay 72 h after transfection with 10 nM *miR-150-5p* and *miR-150-3p*. *$P < 0.0001$. (**B**) Cell movement was assessed by migration assay 48 h after transfection with 10 nM *miR-150-5p* and *miR-150-3p*. *$P < 0.0001$.

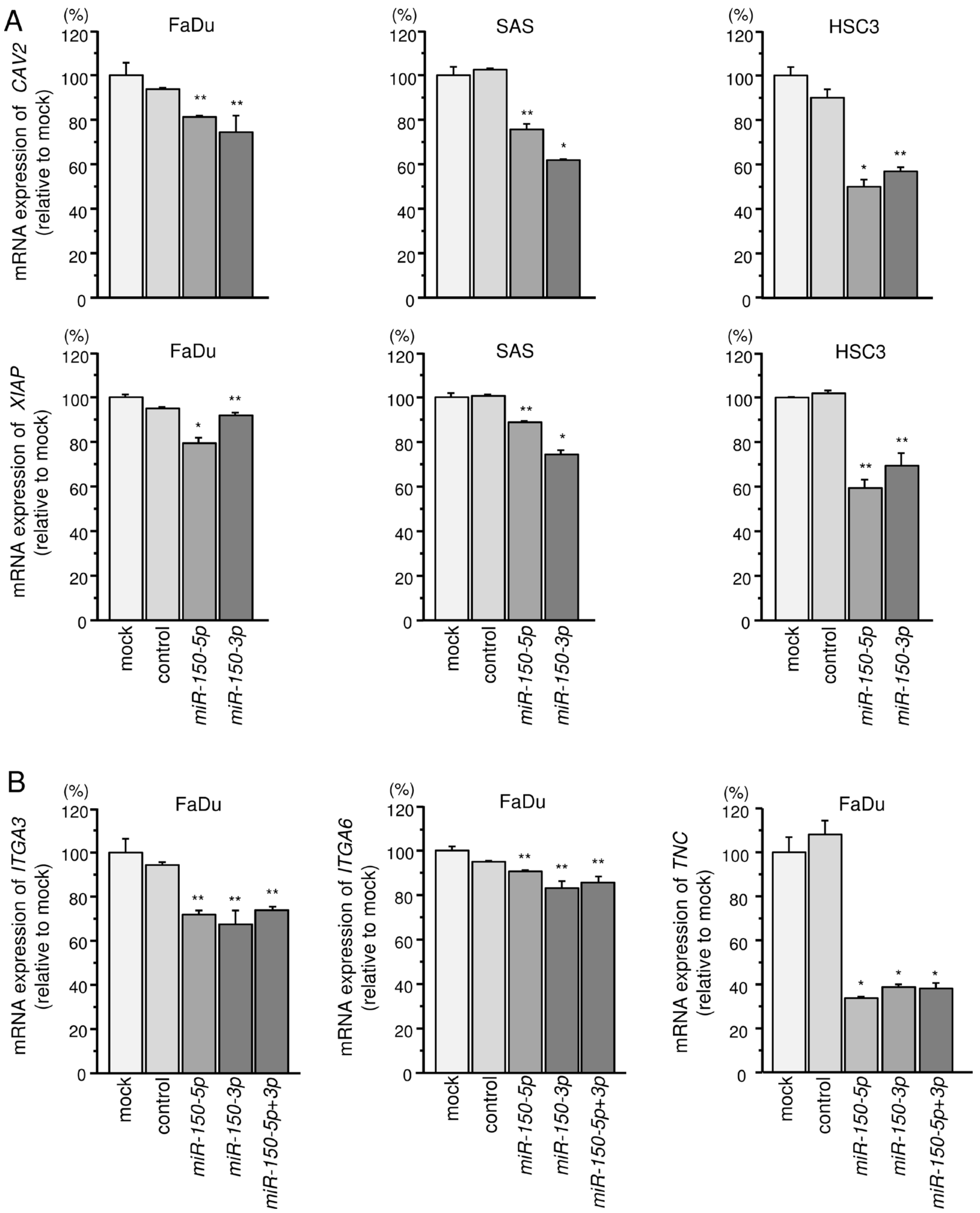

Supplementary Figure 3: Regulation of target gene expression by *miR-150-5p* and *miR-150-3p* and the synergistic effects of *miR-150-5p* and *miR-150-3p* on the genes. (**A**) Expression of *CAV2* and *XIAP* mRNAs 72 h after transfection with 10 nM *miR-150-5p* or *miR-150-3p* into cells. *GUSB* was used as an internal control. $^{*}P \leq 0.0001$, $^{**}P < 0.001$. (**B**) Expression levels of *ITGA3, ITGA6,* and *TNC 72* h after transfection with 10 nM miR-150-5p and/or *miR-150-3p* in HNSCC cells. GUSB was used as an internal control. $^{*}P < 0.0001$, $^{**}P < 0.005$.

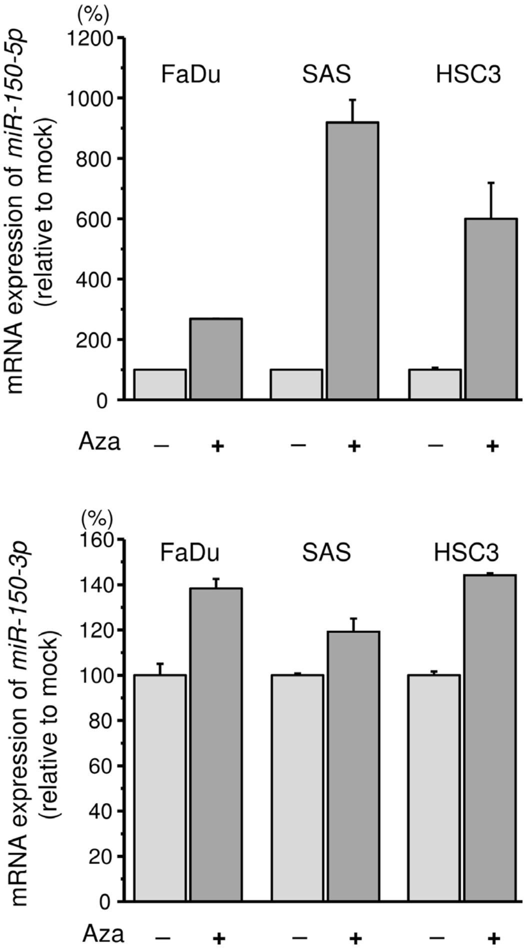

Supplementary Figure 4: Effects of demethylating agent (5-aza-dC) treatment on HNSCC cells. Cell were exposure to 5-aza-dC (1 µM). After 72 hours, expression levels of *miR-150-5p* and *miR-150-3p* were measured by PCR methods. RNU48 was used as normalization.

Supplementary Table 1: Details of sequence reads

Normal epithelia samples	#1		#2		#3		#4		#5		#6	
	Count	(%)	Count	(%)	Count	(%)	Count	(%)	Count	(%)	Count	(%)
Total reads	23679339	100	21946900	100	18443975	100	24404638	100	16558041	100	12180452	100
5’adapter contaminants	7895	0.03	8458	0.04	10466	0.06	6853	0.03	1898	0.01	2339	0.02
3’adapter null	6391072	26.99	7153930	32.6	4964350	26.92	6692336	27.42	1151592	6.95	2543856	20.88
Insert null	79308	0.33	93050	0.42	120598	0.65	88669	0.36	86351	0.52	188043	1.54
Smaller than 15 nt	381930	1.61	476567	2.17	229982	1.25	155685	0.64	437128	2.64	149148	1.22
Longer than 28 nt	6783219	28.65	5494176	25.03	5447702	29.54	6020872	24.67	3193007	19.28	2604380	21.38
Poly A	91	0	141	0	116	0	56	0	184	0	22	0
Clean Reads	10035824	42.38	8720578	39.73	7670761	41.59	11440167	46.88	11687881	70.59	6692664	54.95
**HNSCC samples**	**#1**		**#2**		**#3**		**#4**		**#5**		**#6**	
	Count	(%)	Count	(%)	Count	(%)	Count	(%)	Count	(%)	Count	(%)
Total reads	21362445	100	19174524	100	21930947	100	22141929	100	21717298	100	24845427	100
5’adapter contaminants	6126	0.03	5234	0.03	4818	0.02	4299	0.02	5264	0.02	3473	0.01
3’adapter null	6287386	29.43	6793708	35.43	5858570	26.71	3946545	17.82	4296546	19.78	3015325	12.14
Insert null	89452	0.42	34526	0.18	38111	0.17	55479	0.25	76408	0.35	66033	0.27
Smaller than 15 nt	323869	1.52	269427	1.41	514685	2.35	209630	0.95	328658	1.51	241859	0.97
Longer than 28 nt	5477464	25.64	5773018	30.11	6774839	30.89	6741506	30.45	3255544	14.99	5349161	21.53
Poly A	112	0	46	0	87	0	95	0	37	0	62	0
Clean Reads	9178036	42.96	6298565	32.85	8739837	39.85	11184375	50.51	13754841	63.34	16169514	65.08

Supplementary Table 2: Details of alignment statistics

Normal epithelia samples	#1		#2		#3		#4		#5		#6	
	Count	(%)	Count	(%)	Count	(%)	Count	(%)	Count	(%)	Count	(%)
Clean Reads	10035824	100	8720578	100	7670761	100	11440167	100	11687881	100	6692664	100
Aligned reads	7826695	78	6763551	77.6	6060029	79	9515626	83.2	9598214	82.1	5664692	84.6
- Uniquely matched reads	6051673	60.3	5520277	63.3	4633968	60.4	7667510	67	7566886	64.7	4435139	66.3
- Multiply matched reads	1775022	17.7	1243274	14.3	1426061	18.6	1848116	16.2	2031328	17.4	1229553	18.4
Unaligned reads	2056201	20.5	1782517	20.4	1487183	19.4	1838872	16.1	1940248	16.6	983994	14.7
- No matches found	1806140	18	1595243	18.3	1328557	17.3	1670868	14.6	1715989	14.7	887846	13.3
- Too many matches	250061	2.5	187274	2.1	158626	2.1	168004	1.5	224259	1.9	96148	1.4
Number of locations aligned Uniquely and Multiply matched reads	9004184		7619508		7081118		10886607		11021661		6736173	
**HNSCC samples**	**#1**		**#2**		**#3**		**#4**		**#5**		**#6**	
	Count	(%)	Count	(%)	Count	(%)	Count	(%)	Count	(%)	Count	(%)
Clean Reads	9178036	100	6298565	100	8739837	100	11184375	100	13754841	100	16169514	100
Aligned reads	7114159	77.5	4965770	78.8	6053433	69.3	9145089	81.8	11597592	84.3	13573321	83.9
- Uniquely matched reads	5877644	64	4089163	64.9	4601566	52.7	8043485	71.9	9465308	68.8	11000284	68
- Multiply matched reads	1236515	13.5	876607	13.9	1451867	16.6	1101604	9.8	2132284	15.5	2573037	15.9
Unaligned reads	1933101	21.1	1239192	19.7	2421581	27.7	1965018	17.6	2086889	15.2	2494236	15.4
- No matches found	1714741	18.7	1055087	16.8	1981443	22.7	1790540	16	1912633	13.9	2280134	14.1
- Too many matches	218360	2.4	184105	2.9	440138	5	174478	1.6	174256	1.3	214102	1.3
Number of locations aligned Uniquely and Multiply matched reads	8085627		5690747		7502945		10025234		13663064		15951587	

Supplementary Table 3: Annotation of reads aligned to small RNAs

Normal epithelia samples	#1		#2		#3		#4		#5		#6	
	Count	(%)	Count	(%)	Count	(%)	Count	(%)	Count	(%)	Count	(%)
Total locations	9004184	100	7619508	100	7081118	100	10886607	100	11021661	100	6736173	100
miRNA	7985677	88.69	6665278	87.48	5980706	84.46	9905833	90.99	9722647	88.21	6057813	89.93
scRNA	7304	0.08	5941	0.08	5468	0.08	5291	0.05	7826	0.07	5692	0.08
scRNA_pseudo	3710	0.04	2007	0.03	2972	0.04	1348	0.01	1681	0.02	1104	0.02
snRNA	19246	0.21	40205	0.53	8143	0.11	5765	0.05	9836	0.09	3183	0.05
snRNA_pseudo	170	0.00	723	0.01	59	0.00	31	0.00	30	0.00	15	0.00
snoRNA	766558	8.51	691987	9.08	910311	12.86	780185	7.17	1069951	9.71	586812	8.71
snoRNA_pseudo	37	0.00	34	0.00	41	0.00	60	0.00	167	0.00	49	0.00
tRNA	125358	1.39	107289	1.41	93618	1.32	102256	0.94	81339	0.74	38784	0.58
tRNA_pseudo	1526	0.02	1148	0.02	919	0.01	1236	0.01	1067	0.01	761	0.01
Unknown	94598	1.05	104896	1.38	78881	1.11	84602	0.78	127117	1.15	41960	0.62
**HNSCC samples**	**#1**		**#2**		**#3**		**#4**		**#5**		**#6**	
	**Count**	**(%)**	**Count**	**(%)**	**Count**	**(%)**	**Count**	**(%)**	**Count**	**(%)**	**Count**	**(%)**
Total locations	8085627	100	5690747	100	7502945	100	10025234	100	13663064	100	15951587	100
miRNA	7215729	89.24	5101981	89.65	6428347	85.68	9283993	92.61	12852920	94.07	14811960	92.86
scRNA	19098	0.24	9312	0.16	15689	0.21	7521	0.08	8438	0.06	18489	0.12
scRNA_pseudo	6804	0.08	1585	0.03	4097	0.05	1331	0.01	1600	0.01	2611	0.02
snRNA	11610	0.14	22812	0.40	46239	0.62	18325	0.18	16682	0.12	41466	0.26
snRNA_pseudo	141	0.00	71	0.00	247	0.00	24	0.00	47	0.00	71	0.00
snoRNA	625071	7.73	394915	6.94	657859	8.77	514946	5.14	631060	4.62	899820	5.64
snoRNA_pseudo	72	0.00	68	0.00	134	0.00	94	0.00	215	0.00	240	0.00
tRNA	143407	1.77	104766	1.84	266087	3.55	136841	1.36	89807	0.66	105502	0.66
tRNA_pseudo	1338	0.02	1766	0.03	2813	0.04	1657	0.02	1272	0.01	1481	0.01
Unknown	62357	0.77	53471	0.94	81433	1.09	60502	0.60	61023	0.45	69947	0.44

Supplementary Table 4A: Downregulated miRNAs identified by deep sequencing of HNSCC clinical specimens. See Supplementary_Table_4

Supplementary Table 4B: Upregulated miRNAs identified by deep sequencing of HNSCC clinical specimens. See Supplementary_Table_4
