# Supplementary material for: Deep sequencing-based microRNA expression signatures in head and neck squamous cell carcinoma: dual strands of pre-miR-150 as antitumor miRNAs
Source: Oncotarget. 2017 Mar 17;8(18):30288–304. doi: 10.18632/oncotarget.16327 (PMC5444743; doi:10.18632/oncotarget.16327)
Supplement: Supplementary file 2 [file oncotarget-08-30288-s002.docx]

| Table 2a. Downregulated miRNAs identified by deep sequencing of HNSCC clinical specimens | | | | |  |  |
| --- | --- | --- | --- | --- | --- | --- |
| MicroRNA | Accession No. | Location | Log_2_ fold change | FDR | Normalized read count (Log_2_) | |
|  |  |  |  | (false discovery rate) | Normal | Cancer |
| hsa-miR-375 | MI0000783_1 | 2q35 | -5.463 | 0.0484 | 14.39 | 8.93 |
| hsa-miR-133a-2 | MI0000451_1 | 20q13.33 | -4.535 | 0.3039 | 9.76 | 5.22 |
| hsa-miR-133a-1 | MI0000450_1 | 18q11.2 | -4.502 | 0.3054 | 9.75 | 5.25 |
| hsa-miR-150-3p | MI0000479_1 | 19q13.33 | -3.933 | 0.1521 | 5.70 | 1.76 |
| hsa-miR-1-2 | MI0000437_1 | 18q11.2 | -3.644 | 0.3857 | 14.68 | 11.04 |
| hsa-miR-1-1 | MI0000651_1 | 20q13.33 | -3.636 | 0.3864 | 14.72 | 11.08 |
| hsa-miR-135a-2-5p | MI0000453_1 | 12q23.1 | -3.307 | 0.1744 | 5.86 | 2.55 |
| hsa-miR-135a-1-5p | MI0000452_2 | 3p21.1 | -3.274 | 0.1746 | 5.81 | 2.53 |
| hsa-miR-885-5p | MI0005560_2 | 3p25.3 | -3.160 | 0.1094 | 4.14 | 0.98 |
| hsa-miR-4521 | MI0016887_1 | 17p13 | -3.107 | 0.1898 | 9.08 | 5.97 |
| hsa-miR-150-5p | MI0000479_2 | 19q13.33 | -2.910 | 0.2039 | 13.41 | 10.50 |
| hsa-miR-139-5p | MI0000261_2 | 11q13.4 | -2.863 | 0.0763 | 12.36 | 9.49 |
| hsa-miR-504 | MI0003189_1 | Xq26.3 | -2.745 | 0.1818 | 7.43 | 4.69 |
| hsa-miR-497-5p | MI0003138_2 | 17p13.1 | -2.678 | 0.1496 | 7.89 | 5.21 |
| hsa-miR-99a-3p | MI0000101_2 | 21q21.1 | -2.446 | 0.0749 | 8.17 | 5.72 |
| hsa-miR-100-5p | MI0000102_2 | 11q24.1 | -2.438 | 0.1006 | 17.31 | 14.87 |
| hsa-miR-99a-5p | MI0000101_1 | 21q21.1 | -2.389 | 0.0107 | 18.21 | 15.83 |
| hsa-miR-125b-2-5p | MI0000470_1 | 21q21.1 | -2.373 | 0.0863 | 15.65 | 13.28 |
| hsa-miR-125b-1-5p | MI0000446_2 | 11q24.1 | -2.344 | 0.0882 | 15.64 | 13.29 |
| hsa-miR-338-3p | MI0000814_1 | 17q25.3 | -2.205 | 0.2557 | 6.28 | 4.07 |
| hsa-miR-582-5p | MI0003589_2 | 5q12.1 | -2.095 | 0.1636 | 8.05 | 5.95 |
| hsa-miR-451a | MI0001729_1 | 17q11.2 | -2.063 | 0.3321 | 14.56 | 12.50 |
| hsa-miR-887 | MI0005562_1 | 5p15.1 | -2.053 | 0.2633 | 5.06 | 3.00 |
| hsa-miR-1247-5p | MI0006382_2 | 14q32.31 | -2.034 | 0.2610 | 4.61 | 2.58 |
| hsa-miR-195-5p | MI0000489_2 | 17p13.1 | -2.028 | 0.1470 | 11.16 | 9.13 |
| hsa-miR-144-5p | MI0000460_2 | 17q11.2 | -1.963 | 0.2622 | 8.49 | 6.52 |
| hsa-let-7c | MI0000064_1 | 21q21.1 | -1.931 | 0.0432 | 15.29 | 13.36 |
| hsa-miR-29c-3p | MI0000735_1 | 1q32.2 | -1.929 | 0.3842 | 10.44 | 8.51 |
| hsa-miR-145-5p | MI0000461_1 | 5q32 | -1.927 | 0.1849 | 15.26 | 13.34 |
| hsa-miR-199b-5p | MI0000282_2 | 9q34.11 | -1.896 | 0.2647 | 16.03 | 14.13 |
| hsa-miR-29c-5p | MI0000735_2 | 1q32.2 | -1.872 | 0.2625 | 8.28 | 6.41 |
| hsa-miR-126-5p | MI0000471_1 | 9q34.3 | -1.836 | 0.2607 | 12.79 | 10.95 |
| hsa-miR-29a-3p | MI0000087_1 | 7q32.3 | -1.798 | 0.1147 | 14.70 | 12.91 |
| hsa-miR-664-3p | MI0006442_1 | 1q41 | -1.781 | 0.1149 | 9.84 | 8.06 |
| hsa-miR-125b-1-3p | MI0000446_1 | 11q24.1 | -1.723 | 0.2313 | 8.69 | 6.96 |
| hsa-miR-140-3p | MI0000456_2 | 16q22.1 | -1.721 | 0.1487 | 14.08 | 12.36 |
| hsa-miR-338-5p | MI0000814_2 | 17q25.3 | -1.703 | 0.2685 | 7.45 | 5.75 |
| hsa-miR-486-5p | MI0002470_2 | 8p11.21 | -1.702 | 0.2652 | 11.98 | 10.27 |
| hsa-miR-10b-5p | MI0000267_1 | 2q31.1 | -1.682 | 0.0482 | 17.08 | 15.40 |
| hsa-miR-29a-5p | MI0000087_2 | 7q32.3 | -1.648 | 0.4284 | 4.44 | 2.79 |
| hsa-miR-1468 | MI0003782_1 | Xq11 | -1.611 | 0.2468 | 4.65 | 3.03 |
| hsa-miR-10b-3p | MI0000267_2 | 2q31.1 | -1.585 | 0.1037 | 6.49 | 4.91 |
| hsa-miR-140-5p | MI0000456_1 | 16q22.1 | -1.582 | 0.2556 | 11.22 | 9.64 |
| hsa-miR-195-3p | MI0000489_1 | 17p13.1 | -1.565 | 0.3212 | 8.58 | 7.02 |
| hsa-miR-203 | MI0000283_1 | 14q32.33 | -1.564 | 0.3170 | 17.58 | 16.02 |
| hsa-miR-585 | MI0003592_1 | 5q35.1 | -1.564 | 0.1830 | 6.76 | 5.20 |
| hsa-miR-126-3p | MI0000471_2 | 9q34.3 | -1.560 | 0.1626 | 17.53 | 15.97 |
| hsa-miR-145-3p | MI0000461_2 | 5q32 | -1.552 | 0.2569 | 9.56 | 8.01 |
| hsa-miR-26b-5p | MI0000084_1 | 2q35 | -1.547 | 0.0804 | 15.66 | 14.12 |
| hsa-miR-29b-2-5p | MI0000107_2 | 1q32.2 | -1.531 | 0.1254 | 6.52 | 4.99 |
| hsa-miR-154-5p | MI0000480_1 | 14q32.31 | -1.524 | 0.2568 | 5.64 | 4.11 |
| hsa-miR-146a-5p | MI0000477_1 | 5q33.3 | -1.502 | 0.2794 | 13.38 | 11.88 |
| hsa-miR-26b-3p | MI0000084_2 | 2q35 | -1.498 | 0.0527 | 6.80 | 5.30 |
| hsa-miR-3065-5p | MI0014228_1 | 17q25 | -1.491 | 0.2236 | 8.22 | 6.73 |
| hsa-miR-378a-5p | MI0000786_1 | 5q32 | -1.442 | 0.2016 | 8.68 | 7.24 |
| hsa-miR-664-5p | MI0006442_2 | 1q41 | -1.438 | 0.2612 | 6.71 | 5.27 |
| hsa-miR-342-5p | MI0000805_1 | 14q32.2 | -1.434 | 0.1295 | 7.42 | 5.98 |
| hsa-miR-644b-3p | MI0019134_2 | 20q11.22 | -1.425 | 0.2828 | 5.97 | 4.54 |
| hsa-miR-125a-5p | MI0000469_1 | 19q13.41 | -1.410 | 0.3695 | 14.68 | 13.27 |
| hsa-miR-1255a | MI0006389_1 | 4q24 | -1.397 | 0.2672 | 4.30 | 2.90 |
| hsa-miR-125b-2-3p | MI0000470_2 | 21q21.1 | -1.375 | 0.1042 | 7.40 | 6.02 |
| hsa-miR-143-3p | MI0000459_2 | 5q32 | -1.368 | 0.3447 | 19.41 | 18.04 |
| hsa-miR-26a-2-5p | MI0000750_2 | 12q14.1 | -1.347 | 0.2606 | 18.44 | 17.09 |
| hsa-miR-26a-1-5p | MI0000083_1 | 3p22.2 | -1.345 | 0.2612 | 18.45 | 17.11 |
| hsa-miR-328 | MI0000804_1 | 16q22.1 | -1.299 | 0.2026 | 9.60 | 8.30 |
| hsa-miR-197-3p | MI0000239_2 | 1p13.3 | -1.269 | 0.2220 | 10.84 | 9.57 |
| hsa-miR-30a-3p | MI0000088_1 | 6q13 | -1.262 | 0.3886 | 10.12 | 8.86 |
| hsa-miR-378g | MI0016761_1 | 1p21 | -1.258 | 0.2025 | 7.32 | 6.06 |
| hsa-miR-485-5p | MI0002469_1 | 14q32.31 | -1.239 | 0.2813 | 6.30 | 5.07 |
| hsa-miR-342-3p | MI0000805_2 | 14q32.2 | -1.234 | 0.0529 | 12.11 | 10.88 |
| hsa-miR-199a-2-3p | MI0000281_1 | 1q24.3 | -1.218 | 0.2659 | 16.08 | 14.86 |
| hsa-miR-199a-1-3p | MI0000242_1 | 19p13.2 | -1.217 | 0.2625 | 16.08 | 14.86 |
| hsa-miR-199b-3p | MI0000282_1 | 9q34.11 | -1.217 | 0.2644 | 16.08 | 14.86 |
| hsa-miR-378a-3p | MI0000786_2 | 5q32 | -1.212 | 0.1371 | 15.89 | 14.67 |
| hsa-miR-186-5p | MI0000483_2 | 1p31.1 | -1.170 | 0.1098 | 12.95 | 11.78 |
| hsa-miR-146a-3p | MI0000477_2 | 5q33.3 | -1.160 | 0.4081 | 4.07 | 2.91 |
| hsa-miR-574-3p | MI0003581_2 | 4p14 | -1.153 | 0.2135 | 11.58 | 10.43 |
| hsa-miR-378i | MI0016902_1 | 22q13.2 | -1.153 | 0.2594 | 4.38 | 3.23 |
| hsa-miR-30e-3p | MI0000749_2 | 1p34.2 | -1.138 | 0.2616 | 11.47 | 10.33 |
| hsa-miR-200b-5p | MI0000342_1 | 1p36.33 | -1.100 | 0.2299 | 9.24 | 8.14 |
| hsa-miR-3065-3p | MI0014228_2 | 17q25 | -1.080 | 0.2784 | 5.47 | 4.39 |
| hsa-miR-3912 | MI0016416_1 | 5q35.1 | -1.066 | 0.4073 | 4.99 | 3.93 |
| hsa-let-7e-3p | MI0000066_2 | 19q13.41 | -1.064 | 0.2466 | 6.42 | 5.36 |
| hsa-miR-326 | MI0000808_1 | 11q13.4 | -1.061 | 0.2804 | 6.10 | 5.04 |
| hsa-let-7b-3p | MI0000063_2 | 22q13.31 | -1.055 | 0.3603 | 8.28 | 7.23 |
| hsa-miR-874 | MI0005532_1 | 5q31.2 | -1.046 | 0.3647 | 8.80 | 7.76 |
| hsa-miR-30b-5p | MI0000441_2 | 8q24.22 | -1.004 | 0.0890 | 13.59 | 12.58 |
| hsa-miR-2110 | MI0010629_1 | 10q25.3 | -0.977 | 0.2023 | 7.55 | 6.58 |
| hsa-miR-628-5p | MI0003642_2 | 15q21.3 | -0.974 | 0.3709 | 6.09 | 5.12 |
| hsa-miR-30c-1-5p | MI0000736_1 | 1p34.2 | -0.951 | 0.2704 | 14.51 | 13.56 |
| hsa-miR-30c-2-5p | MI0000254_2 | 6q13 | -0.947 | 0.2705 | 14.48 | 13.53 |
| hsa-miR-10a-5p | MI0000266_2 | 17q21.32 | -0.940 | 0.3895 | 15.98 | 15.04 |
| hsa-miR-487b | MI0003530_1 | 14q32.31 | -0.908 | 0.2734 | 6.26 | 5.36 |
| hsa-miR-30c-1-3p | MI0000736_2 | 1p34.2 | -0.904 | 0.3419 | 6.61 | 5.71 |
| hsa-miR-625-5p | MI0003639_1 | 14q23.3 | -0.901 | 0.4267 | 4.82 | 3.92 |
| hsa-miR-942 | MI0005767_1 | 1p13.1 | -0.879 | 0.0563 | 5.30 | 4.42 |
| hsa-let-7g-5p | MI0000433_2 | 3p21.1 | -0.864 | 0.1955 | 18.05 | 17.18 |
| hsa-miR-502-3p | MI0003186_2 | Xp11.23 | -0.840 | 0.0780 | 9.74 | 8.90 |
| hsa-miR-379-5p | MI0000787_1 | 14q32.31 | -0.822 | 0.3836 | 10.64 | 9.82 |
| hsa-miR-576-5p | MI0003583_1 | 4q25 | -0.800 | 0.2818 | 7.31 | 6.51 |
| hsa-miR-625-3p | MI0003639_2 | 14q23.3 | -0.791 | 0.2592 | 7.52 | 6.72 |
| hsa-miR-660-5p | MI0003684_1 | Xp11.23 | -0.787 | 0.3009 | 11.20 | 10.42 |
| hsa-miR-374a-5p | MI0000782_2 | Xq13.2 | -0.783 | 0.2790 | 10.76 | 9.98 |
| hsa-miR-378c | MI0015825_1 | 10q26.3 | -0.769 | 0.4066 | 11.07 | 10.30 |
| hsa-miR-148a-3p | MI0000253_1 | 7p15.2 | -0.712 | 0.3867 | 18.30 | 17.59 |
| hsa-miR-191-5p | MI0000465_2 | 3p21.31 | -0.704 | 0.3450 | 14.72 | 14.02 |
| hsa-miR-30b-3p | MI0000441_1 | 8q24.22 | -0.674 | 0.2607 | 5.90 | 5.23 |
| hsa-miR-3158-2-3p | MI0014187_1 | 10q24.3 | -0.607 | 0.2657 | 6.08 | 5.47 |
| hsa-miR-200b-3p | MI0000342_2 | 1p36.33 | -0.593 | 0.3704 | 16.88 | 16.29 |
| hsa-miR-148a-5p | MI0000253_2 | 7p15.2 | -0.592 | 0.4111 | 10.55 | 9.96 |
| hsa-miR-3158-1-3p | MI0014186_2 | 10q24.3 | -0.589 | 0.2550 | 6.10 | 5.51 |
| hsa-miR-320a | MI0000542_1 | 8p21.3 | -0.571 | 0.2265 | 14.29 | 13.72 |
| hsa-miR-374b-5p | MI0005566_2 | Xq13.2 | -0.561 | 0.1507 | 12.35 | 11.79 |
| hsa-miR-16-2-5p | MI0000115_1 | 3q25.33 | -0.515 | 0.3798 | 13.21 | 12.70 |
| hsa-miR-16-1-5p | MI0000070_2 | 13q14.2 | -0.505 | 0.3848 | 13.22 | 12.71 |
| hsa-miR-141-3p | MI0000457_2 | 12p13.31 | -0.402 | 0.4244 | 10.99 | 10.59 |
|  |  |  |  |  |  |  |
| Table 2b. Upregulated miRNAs identified by deep sequencing of HNSCC clinical specimens | | | | |  |  |
| MicroRNA | Accession No. | Location | Log_2_ fold change | FDR | Normalized read count (Log_2_) | |
|  |  |  |  | (false discovery rate) | Normal | Cancer |
| hsa-miR-615-3p | MI0003628_2 | 12q13.13 | 5.145 | 0.1353 | 2.82 | 7.97 |
| hsa-miR-196b-5p | MI0001150_2 | 7p15.2 | 3.075 | 0.0138 | 5.91 | 8.98 |
| hsa-miR-187-3p | MI0000274_1 | 18q12.2 | 2.383 | 0.2044 | 5.33 | 7.71 |
| hsa-miR-503 | MI0003188_1 | Xq26.3 | 2.165 | 0.1995 | 5.87 | 8.03 |
| hsa-miR-542-3p | MI0003686_1 | Xq26.3 | 2.115 | 0.1023 | 6.80 | 8.91 |
| hsa-miR-450b-5p | MI0005531_2 | Xq26.3 | 2.057 | 0.1723 | 7.03 | 9.09 |
| hsa-miR-34c-5p | MI0000743_1 | 11q23.1 | 1.820 | 0.4197 | 9.72 | 11.54 |
| hsa-miR-450a-2-5p | MI0003187_2 | Xq26.3 | 1.787 | 0.1213 | 7.12 | 8.91 |
| hsa-miR-424-3p | MI0001446_1 | Xq26.3 | 1.783 | 0.3713 | 6.13 | 7.91 |
| hsa-miR-450a-1-5p | MI0001652_1 | Xq26.3 | 1.782 | 0.1227 | 7.12 | 8.90 |
| hsa-miR-7a-1-5p | MI0000263_2 | 9q22.32 | 1.771 | 0.0596 | 13.48 | 15.25 |
| hsa-miR-7a-3-5p | MI0000265_1 | 22q13.31 | 1.763 | 0.0566 | 13.43 | 15.19 |
| hsa-miR-7a-2-5p | MI0000264_1 | 11q24.1 | 1.759 | 0.0542 | 13.44 | 15.20 |
| hsa-miR-9-3-5p | MI0000468_1 | 15q26.1 | 1.692 | 0.2772 | 9.88 | 11.57 |
| hsa-miR-9-1-5p | MI0000466_2 | 1q22 | 1.687 | 0.2784 | 9.85 | 11.54 |
| hsa-miR-9-2-5p | MI0000467_2 | 5q14.3 | 1.687 | 0.2769 | 9.85 | 11.54 |
| hsa-miR-21-5p | MI0000077_1 | 17q23.1 | 1.597 | 0.1526 | 19.70 | 21.30 |
| hsa-miR-21-3p | MI0000077_2 | 17q23.1 | 1.548 | 0.1204 | 11.11 | 12.66 |
| hsa-miR-4664-3p | MI0017294_1 | 8q24.3 | 1.455 | 0.2223 | 4.57 | 6.03 |
| hsa-miR-424-5p | MI0001446_2 | Xq26.3 | 1.405 | 0.2095 | 7.00 | 8.40 |
| hsa-miR-25-5p | MI0000082_2 | 7q22.1 | 1.398 | 0.1087 | 6.10 | 7.50 |
| hsa-miR-147b | MI0005544_1 | 15q21.1 | 1.364 | 0.2026 | 5.43 | 6.79 |
| hsa-miR-708-3p | MI0005543_1 | 11q14.1 | 1.297 | 0.4250 | 8.49 | 9.79 |
| hsa-miR-1301 | MI0003815_1 | 2p23 | 1.158 | 0.2603 | 6.99 | 8.15 |
| hsa-miR-548k | MI0006354_1 | 11q13.3 | 1.144 | 0.3456 | 6.79 | 7.94 |
| hsa-miR-671-5p | MI0003760_1 | 7q36.1 | 1.137 | 0.1549 | 7.76 | 8.89 |
| hsa-miR-2355-3p | MI0015873_1 | 2q33 | 1.135 | 0.2624 | 3.84 | 4.97 |
| hsa-miR-550a-2-5p | MI0003601_1 | 7p14.3 | 1.130 | 0.1268 | 3.64 | 4.77 |
| hsa-miR-550a-1-5p | MI0003600_1 | 7p14.3 | 1.130 | 0.1295 | 3.64 | 4.77 |
| hsa-miR-944 | MI0005769_1 | 3q28 | 1.062 | 0.3709 | 9.37 | 10.43 |
| hsa-miR-106b-3p | MI0000734_1 | 7q22.1 | 1.036 | 0.2602 | 10.61 | 11.64 |
| hsa-miR-5001-3p | MI0017867_1 | 2q37.1 | 1.033 | 0.3864 | 3.84 | 4.88 |
| hsa-miR-877-5p | MI0005561_1 | 6p21.33 | 1.030 | 0.1822 | 5.90 | 6.93 |
| hsa-miR-18a-5p | MI0000072_1 | 13q31.3 | 0.981 | 0.1865 | 7.25 | 8.23 |
| hsa-miR-335-3p | MI0000816_2 | 7q32.2 | 0.853 | 0.4126 | 9.44 | 10.29 |
| hsa-miR-92b-3p | MI0003560_2 | 1q22 | 0.791 | 0.2816 | 9.68 | 10.47 |
| hsa-miR-149-5p | MI0000478_1 | 2q37.3 | 0.763 | 0.1446 | 10.88 | 11.64 |
| hsa-miR-455-5p | MI0003513_1 | 9q32 | 0.753 | 0.2654 | 11.66 | 12.41 |
| hsa-miR-93-3p | MI0000095_1 | 7q22.1 | 0.654 | 0.1612 | 6.52 | 7.17 |
| hsa-miR-25-3p | MI0000082_1 | 7q22.1 | 0.643 | 0.3837 | 13.82 | 14.47 |
| hsa-miR-454-3p | MI0003820_1 | 17q22 | 0.627 | 0.2798 | 8.73 | 9.36 |
| hsa-miR-330-5p | MI0000803_2 | 19q13.32 | 0.593 | 0.3877 | 6.56 | 7.15 |
| hsa-miR-93-5p | MI0000095_2 | 7q22.1 | 0.545 | 0.4288 | 13.03 | 13.57 |
| hsa-miR-152 | MI0000462_1 | 17q21.32 | 0.387 | 0.3723 | 13.62 | 14.01 |
| hsa-miR-3934 | MI0016590_1 | 6p21.3 | 0.237 | 0.3291 | 5.63 | 5.87 |
